# Meta-QTL analysis explores the key genes, especially hormone related genes, involved in the regulation of grain water content and grain dehydration rate in maize

**DOI:** 10.1186/s12870-022-03738-y

**Published:** 2022-07-16

**Authors:** Wei Wang, Zhaobin Ren, Lu Li, Yiping Du, Yuyi Zhou, Mingcai Zhang, Zhaohu Li, Fei Yi, Liusheng Duan

**Affiliations:** 1 State Key Laboratory of Plant Physiology and Biochemistry, Engineering Research Center of Plant Growth Regulator, Ministry of Education &College of Agronomy and Biotechnology, China Agricultural University, No.2 Yuanmingyuan West Road, Haidian, Beijing, 100193 China; 2 College of Plant Science and Technology, Beijing University of Agriculture, Beijing, 102206 China

**Keywords:** Grain water content, Grain dehydration rate, QTL, Meta-analysis, Hormone

## Abstract

**Background:**

Low grain water content (GWC) at harvest of maize (*Zea mays* L.) is essential for mechanical harvesting, transportation and storage. Grain drying rate (GDR) is a key determinant of GWC. Many quantitative trait locus (QTLs) related to GDR and GWC have been reported, however, the confidence interval (CI) of these QTLs are too large and few QTLs has been fine-mapped or even been cloned. Meta-QTL (MQTL) analysis is an effective method to integrate QTLs information in independent populations, which helps to understand the genetic structure of quantitative traits.

**Results:**

In this study, MQTL analysis was performed using 282 QTLs from 25 experiments related GDR and GWC. Totally, 11 and 34 MQTLs were found to be associated with GDR and GWC, respectively. The average CI of GDR and GWC MQTLs was 24.44 and 22.13 cM which reduced the 57 and 65% compared to the average QTL interval for initial GDR and GWC QTL, respectively. Finally, 1494 and 5011 candidate genes related to GDR and GWC were identified in MQTL intervals, respectively. Among these genes, there are 48 genes related to hormone metabolism.

**Conclusions:**

Our studies combined traditional QTL analyses, genome-wide association study and RNA-seq to analysis major locus for regulating GWC in maize.

**Supplementary Information:**

The online version contains supplementary material available at 10.1186/s12870-022-03738-y.

## Background

Maize (*Zea mays* L.) is one of the most important crops in the world and serves as an important source of feed, food, energy and industrial raw materials. However, the excessively high grain water content (GWC) during maize harvest severely limits the efficiency of mechanized harvesting and increases the cost of drying and storage in China [1]. Especially in the Huang-Huai-Hai region of China, farmers usually delay harvesting to reduce GWC. The measure delays the planting time of the next crops, and greatly affects the annual yield [2]. Grain drying rate (GDR) is a key determinant of GWC [3]. Therefore, increasing GDR and reducing GWC at harvest have become the significant goals of modern maize breeding. The GWC at maturity in maize was mainly due to genetic factors [4], although it is also influenced by environmental factors [4]. Consequently, increasing GDR and reducing GWC from a genetic point of view are of great significance for increasing the maize yield in Wheat-Maize Rotation System of Huang-Huai-Hai region.

Dehydration of maize grains is a complex process involving many physiological and biochemical changes. Studies have shown that maize varieties with fast GDR have faster filling speed. The variation of GWC and GDR has significant changes in the expression of genes related starch biosynthesis [5]. In addition, the growth and development of maize grains is regulated by a variety of plant hormones. The dehydration process of maize grains was likely to be affected by the programmed cell death (PCD) process [6]. Ethylene (ETH) and abscisic acid (ABA) were key regulators of PCD during plant development [7]. ETH treatment could induce early endosperm DNA fragmentation in wheat and maize, while the use of substances that inhibit ETH synthesis, such as 2-aminoethoxyvinylglycine (AVG) treatment, could significantly delay endosperm DNA fragmentation [7]. And ABA can inhibit the occurrence of PCD caused by ETH [7]. The dead cells that had experienced PCD could promote the release of auxin (IAA) [8]. Gibberellin (GA) could also regulate the PCD process of the aleurone layer cells in the grain [9]. Increasing the level of ABA in maize grains had also been shown to accelerate the process of dehydration in the grains [10]. Therefore, many hormones may play a role in the process of grain dehydration. The candidate genes were widely present in the metabolism pathways of various hormones. However, it was still unclear whether there were other hormones and which genes related to hormones played a role in this process.

Since the 1990s, there have been many studies on quantitative trait loci (QTL) mapping related to GDR and GWC [11–16]. Sala et al. identified 3 QTLs related GDR and 6 QTLs related GWC through 181 F_2:3_ one-hybrid families in 2006 [12], accounting for 7.00-14.20% and 10.40-19.70% of the phenotypic variation, respectively [12]. Liu et al. used 232 recombinant inbred lines (RILs) to detect 9 QTLs related GDR in 2010, which accounted for 5.77-13.63% of the total GDR variation [11]. Wang et al. used 280 RILs to identify 14 QTLs related GDR in 2012, and each QTL can explain 5.05-16.28% of the total GDR variation [13]. Li et al. used 258 RILs to detect 35 QTLs related GDR and 40 QTLs related GWC during 4 grain filling stages in 2014 [14]. Li et al. used a recombined inbred population of 242 families to detect 4 QTLs related to GWC in 2019, accounting for 2.19-9.28% of the phenotypic variation [16]. Among them, qGm9-1 overlapped with the QTL9/34 confidence interval (CI) of GWC located by study result of Sala et al. [12], so this QTL may have greater research and utilization value. Up to now, QTLs had been reported on all 10 pairs of maize chromosomes related to GDR and GWC. However, the results of the mapping showed great differences with the different test materials and locations. So far, few of the detected QTLs has been widely recognized by everyone.

In order to make the results of QTL mappings that have been obtained can be efficiently applied to maize breeding practice, we need to summarize and integrate them to screen out QTLs with high effects and small CI. Meta-QTL (MQTL) analysis is a good integrated method. It had been used to estimate the CI of many traits of QTL in many crops [17–20]. In 2012, Sala et al. summarized 184 QTLs related GWC and performed MQTL analysis on GWC of maize, 34 MQTLs related to GWC were obtained [21]. In the same year, Xiang et al. used 96 QTLs from 12 experiments for a MQTL analysis and 44 MQTLs were identified [17]. However, these two studies only performed MQTL analysis for GWC-related QTLS, and did not perform MQTL analysis for GDR related QTLs. Moreover, in both studies, only GWC-related MQTLs with smaller CI were obtained, but genes in the obtained MQTLs were not analyzed.

As 10 years later, some more literatures about QTL mapping related to GDR and GWC have been published [11, 13, 14, 16]. Compared with the articles related GWC published by Sala et al. and Xiang et al. in 2012, we removed some QTLs that lacked sufficient information, and added 87 QTLs from 4 latest articles [14, 16, 22, 23]. In total, for GWC, we summarized 195 QTLs and performed MQTL analysis. For GDR, we conducted a MQTL analysis on the 87 QTLs from 7 experiments [11–15, 22, 23]. Gene ontology (GO) enrichment analysis and RNA-seq data were also used to analyze the genes in the mapped MQTLs. It was believed that our research results could provide guidance for future research and cloning of genes related to GDR and GWC.

## Results

### Main features of consensus maps and initial QTLs related GDR and GWC

**Fig. 1:**
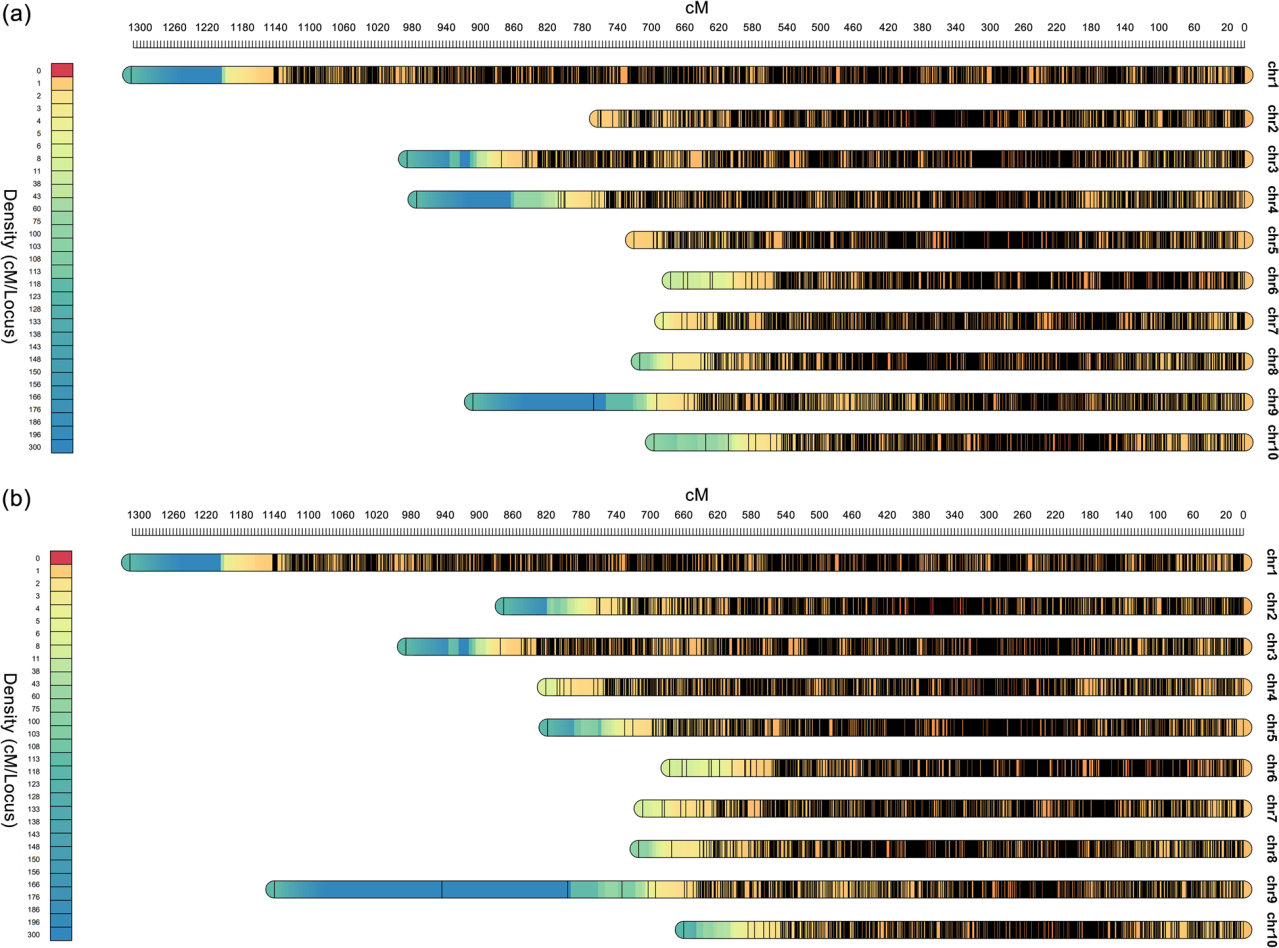
Overview of genome-wide Marker density in the consensus maps related GDR (**a**) and GWC (**b**). The ordinate shows the genetic distance along each of the 10 linkage groups corresponding to the maize genome

Twenty-five experiments were found to present QTL and mapping data for GDR and GWC as of December of 2021 (Table 1). Only 14 of those provided sufficient information on mapping and QTL characteristics to carry out map projections and MQTL analysis. For the remain 11 experiments, we could not find their genetic map, but the genetic markers or physical position at both ends of the QTLs mapped to them could be found in the final integrated linkage map, so they also could be used for the following research. The integrated linkage maps associated GDR and GWC were assembled from 14 individual linkage maps and one previously published reference map IBM2 2008 Neighbors (https://www.maizegdb.org/data_center/map). The integrated linkage maps contained 20,301 (related GDR) and 20,376 (related GWC) markers, respectively (Supplementary Data 1). The total length of 2 integrated linkage maps is 8427 cM (related GDR) and 8702 cM (related GWC), respectively (Supplementary Data 1). The densities of the average of all groups for the integrated linkage groups were 2.41 and 2.34 markers per cM for GDR and GWC, respectively (Fig. 1, Supplementary Data 1).

**Table 1:** Summary of QTL mapping experiments included in MQTL analysis related to GDR and GWC

Reference	Parents of population^**a**^	Type/s of population/s	NO. of environments	Population size/s^**b**^	Methods used^**c**^	NO. of QTLs	Related traits
Sala et al. 2006 [12]	(A, B)	F_2:3_	2	181	IM	3	GDR
Caplelle et al. 2010	(F2, F252)	F_3:4_	1	322	CIM	3	GDR
Liu et al. 2010 [11]	(Ji846, Ye3189)	F_7_	2	232	CIM	9	GDR
Wang et al. 2012 [13]	(Ji846, Ye3189)	F_7:8_	2	280	CIM	14	GDR
Li et al. 2014 [14]	(N04, Dan232)	RIL	2	258	CIM	35	GDR
Yin et al. 2020 [23]*	(DH1M, T877)	RIL	3	208	CIM	6	GDR
Liu et al. 2020 [18]*	(844, 807)	RIL	3	84, 119, 117	CIM	17	GDR
Stuber et al. 1992	(B73, Mo17)	BC_1_	6	264	IM	8	GWC
Beavis et al. 1994	(B73, Mo17)	TC	5	112	IM	10	GWC
Ragot et al. 1995	Population H	F_2_	5	400	SPA and IM	9	GWC
Melchinger et al. 1998	(KW1265, D146)	TC	2	380	CIM	3	GWC
Austin et al. 2000	(Mo17, H99)	F_2:3_	8	194	CIM	18	GWC
Austin et al. 2000 [24]	(Mo17, H99)	F_6:8_	8	186	CIM	12	GWC
Ho et al. 2002	(RD6502, RD3013)	BC_2_TC	6	204	SPA and IM	6	GWC
Moreau et al. 2004	(F2, F252)	F_3:4_	20	300	CIM	26	GWC
Blanc et al. 2006	(DE, F283, F810, F9005), MBS847	F_2:3_	10	150	CIM	13	GWC
Sala et al. 2006 [12]	(A, B)	F_2:3_	2	181	IM	6	GWC
Frascaroli et al. 2007	(B73, H99)	TC	3	142	CIM	14	GWC
Frascaroli et al. 2007	(B73, H99)	TC	3	284	CIM	4	GWC
Robertson et al. 2007	(FR1064, GE440)	BC_1_F_1:2_	8	213	CIM	5	GWC
Capelle et al. 2010 [15]	(F2, F252)	F_3:4_	1	322	CIM	12	GWC
Li et al. 2014 [14]	(N04, Dan232)	RIL	2	258	CIM	40	GWC
Li et al. 2019 [16]	(RA, M53)	RIL	2	242	CIM	4	GWC
Yin et al. 2020 [23]*	(DH1M, T877)	RIL	3	208	CIM	19	GWC
Liu et al. 2020 [18]*	(844, 807)	RIL	3	84, 119, 117	CIM	31	GWC

^a^Inbred lines between parentheses are the original parents of the population, followed by the tester inbred line/s. For proprietary reasons, progenitor lines in Sala et al. (2006) [12] are arbitrarily denominated A and B (Sala et al. 2006) [12]

^b^ Three population numbers appeared in some studies, and the genetic map used in the final study was integrated after mapping these three populations separately

^c^The method used for QTL mapping. The “IM” represents interval mapping, the “CIM” represents composite interval mapping and the “SPA” represents single-point analysis

*The marked “*” represents the QTL mapping experiment with SNP markers

**Fig. 2:**
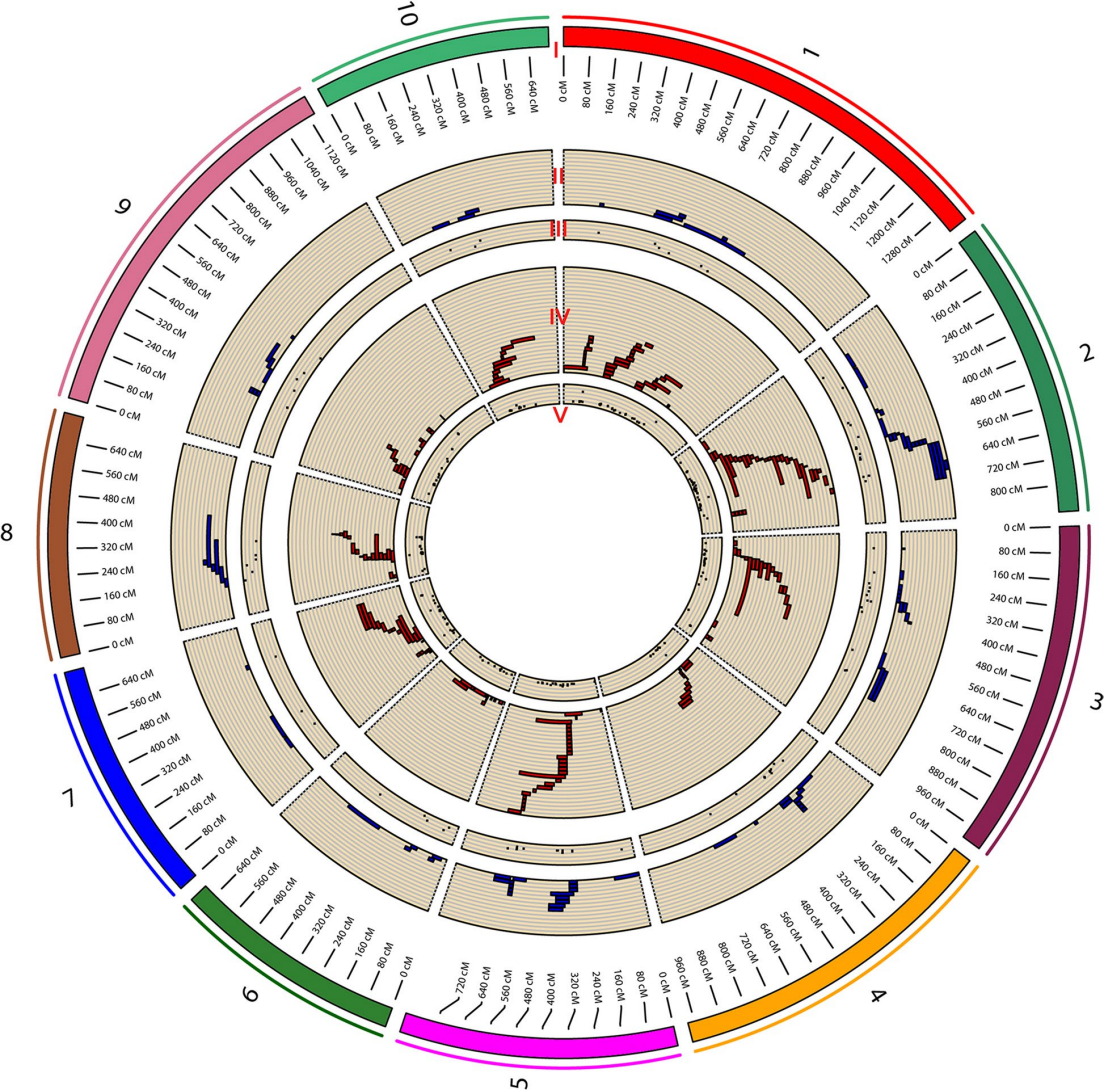
Circos diagram for initial QTLs related GDR and GWC. I, colored bars showing the ten maize chromosomes and chro-mosomal positions (cM) presented along the chromosomes. II, distribution of the initial QTLs related GDR on the ten chromosomes of maize (blue). III, proportion of phenotypic variance explained (*R*^*2*^) for each initial QTLs related GDR. IV, distribution of the initial QTLs related GWC on the ten chromosomes of maize (red). V, proportion of phenotypic variance explained (*R*^*2*^) for each initial QTLs related GWC

A total of 282 QTLs, including 87 related GDR and 195 related GWC (Fig. 2, Supplementary Data 2), were used to perform the MQTL analysis. The number of initial QTLs for GDR and GWC and their distribution on the maize chromosomes were shown in Fig. 2 and Supplementary Fig. 1a. The results indicated a non-random distribution of QTLs within the maize genome. The individual QTLs were scattered unevenly across chromosomes. For GDR and GWC, chromosome 2 showed the highest number of QTLs (18 QTLs for GDR and 32 QTLs for GWC). Chromosome 10, with 3 QTLs for GDR and 10 QTLs for GWC, had the lowest number of QTLs (Fig. 2, Supplementary Fig. 1a).

In addition, for GDR, the CI of a single QTL ranged from 4.42 to 295.95 with an average of 56.48 cM (Supplementary Fig. 1b). The phenotypic variation explained (PVE) by a single QTL ranged from 1 to 29% (with an average of 9%), with most of the QTLs exhibiting 5 < PVE ≤ 20% (Supplementary Fig. 1c). For GWC, the CI of a single QTL ranged from 0.74 to 475.76 with an average of 63.67 cM (Supplementary Fig. 1b). The PVE by a single QTL ranged from 1 to 90% (with an average of 14%), with most of the QTLs exhibiting 5 < PVE ≤ 20% (Supplementary Fig. 1c).

### MQTL analysis related GDR and GWC

The results of MQTL analysis were listed in Supplementary Table 1. In total, 21 MQTLs related GDR and 47 MQTLs related GWC were identified by means of meta-analysis, each MQTL contained 1 to 16 initial QTLs (Supplementary Table 1).

**Fig. 3:**
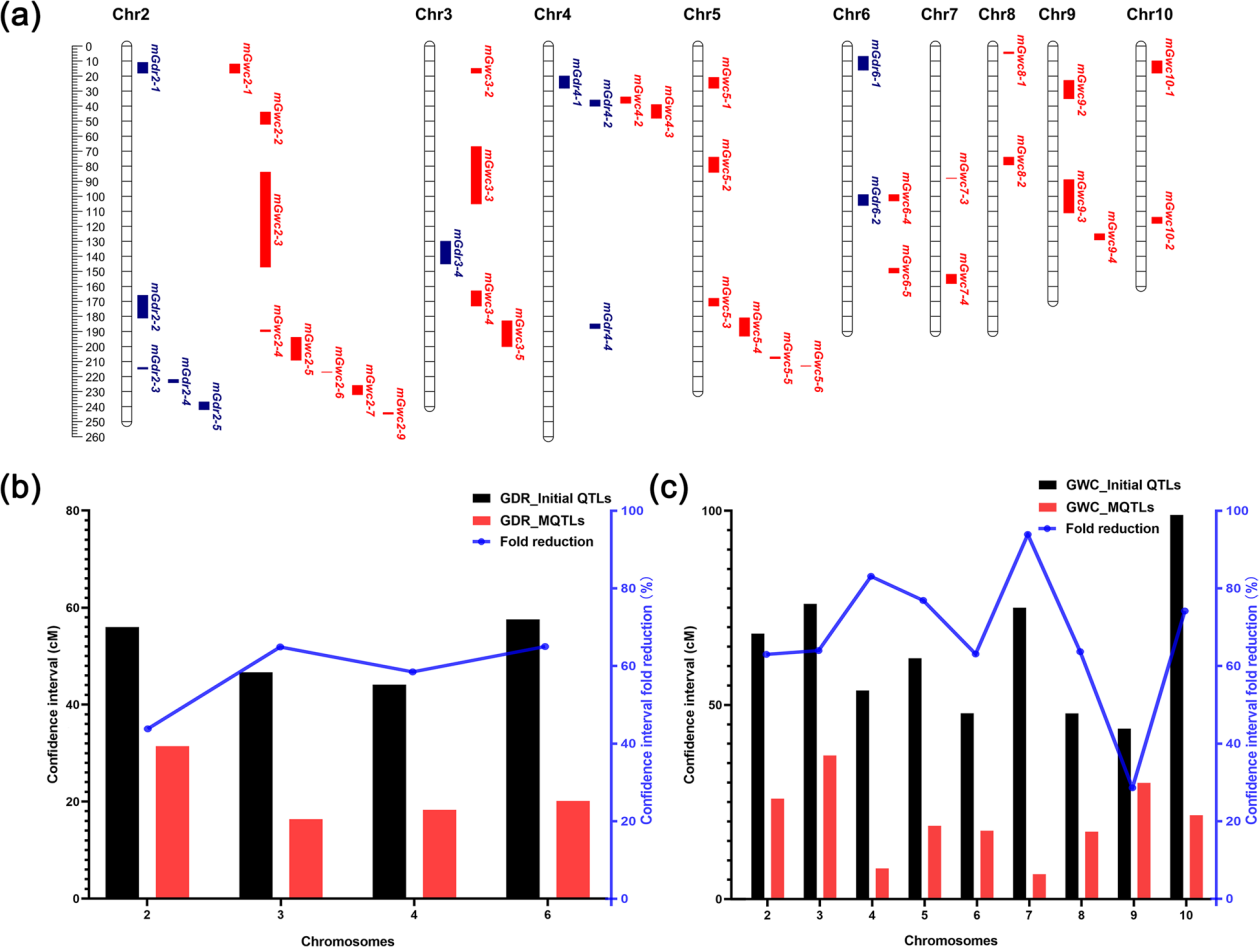
Information of MQTLs affecting GDR and GWC. **a**, positions of MQTLs affecting GDR and GWC in B73 (AGPv4). Dis-tances on the map are in Mb. The MQTLs related to GDR are marked in blue, and those related to GWC are marked in red. **b** and **c**, the fold reduction in CI of QTLs after MQTL analysis related GDR (**b**) and GWC (**c**). The fold reduction is used here (CI of initial QTLs - CI of MQTLs)/CI of initial QTLs

MQTLs with a large number of initial QTLs detected in multiple environments were considered to be more reliable and stable MQTLs independent of phenotypic environment and genetic background. Therefore, in this study, MQTLs formed from the initial QTLs detected in two or more independent experiments were selected as the final candidate MQTLs. Finally, for GDR, 11 MQTLs were identified (Fig. 3a, Table 2). The average CI of these MQTLs was 24.44 cM, with a maximum and minimum value of 70.84 cM (mGdr2-5 on linkage group 2) and 7.43 cM (mGdr2-3 on linkage group 2), respectively. The MQTL analysis reduced the average interval 57% compared to the average QTL interval for initial QTL (56.48 cM). There were significant differences in the mean CI of multiple QTLs among different chromosomes. For example, the mean CI for MQTLs on chromosome 6 was reduced by 65%, but only reduced by 44% on chromosome 2 (Fig. 3b). 11 MQTLs distributed on chromosomes 2, 3, 4 and 6 with 5, 1, 3 or 2 MQTLs, respectively. The mGdr2-3 with the CI of 7.43 cM on chromosome 2 integrated 6 initial QTLs, the mGdr2-4 with the CI of 13.87 cM on chromosome 2 integrated 9 initial QTLs and mGdr4-2 with the CI of 10.92 cM on chromosome 4 integrated 7 initial QTLs. The remaining 8 MQTLs are all aggregated from less than 5 initial QTLs (Table 2).

**Table 2:** Information of GDR and GWC MQTLs we got through MQTL analysis

MQTL	Chromosome	Position (cM)	PVE (%)	CI (cM)	Left Physical Position (bp)	Right Physical Position (bp)	QTL Integrated	NO. of Genes	NO. of Experiments Involved.
mGdr2-1	2	151.15	15.78	53.14	10,982,223	17,884,593	2	232	2
mGdr2-2	2	380.61	10.98	11.94	166,122,155	181,169,734	3	256	3
mGdr2-3	2	502.05	7.64	7.43	214,385,071	215,364,994	6	32	3
mGdr2-4	2	561.27	8.68	13.87	222,366,098	224,367,093	9	69	3
mGdr2-5	2	668.97	10.15	70.84	237,260,984	241,600,111	4	158	2
mGdr3-4	3	312.46	10.05	16.4	129,690,026	145,298,038	3	245	3
mGdr4-1	4	214.74	5.27	32.12	19,980,502	28,015,268	2	131	2
mGdr4-2	4	258.55	7.89	10.92	35,865,238	40,230,939	7	96	2
mGdr4-4	4	460.24	4.67	11.9	184,544,196	187,853,075	2	82	2
mGdr6-1	6	59.17	9.19	20.88	6,930,202	15,745,036	2	65	2
mGdr6-2	6	166.72	6.81	19.42	99,067,473	105,548,698	2	128	2
mGwc2-1	2	155.94	6.78	41.93	12,427,429	17,785,263	4	175	4
mGwc2-2	2	301.82	7.97	22.73	44,127,325	51,838,656	7	155	6
mGwc2-3	2	347.41	9.37	18.91	83,753,787	146,866,826	8	516	5
mGwc2-4	2	391.63	9.29	13.1	188,916,996	189,613,605	11	15	7
mGwc2-5	2	434.325	8.77	56.88	194,221,855	208,614,777	9	396	6
mGwc2-6	2	515.9	14.62	4.67	216,546,581	216,742,980	6	4	4
mGwc2-7	2	591.77	4.90	30.23	225,740,064	231,716,906	8	130	5
mGwc2-9	2	729.37	23.92	18.71	244,082,956	244,987,304	5	20	2
mGwc3-2	3	167.4	11.54	8.17	14,756,549	18,158,448	9	76	5
mGwc3-3	3	246.92	13.88	17.67	66,877,732	105,421,272	9	216	3
mGwc3-4	3	379.88	16.36	30.34	163,435,936	172,954,011	4	165	3
mGwc3-5	3	496.27	20.80	91.86	182,863,176	199,730,257	2	454	2
mGwc4-2	4	251.93	16.54	3.5	34,288,458	38,196,068	7	70	3
mGwc4-3	4	268.21	8.27	12.33	39,303,177	48,419,875	5	147	3
mGwc5-1	5	227.47	5.51	9.9	21,441,502	28,139,219	2	136	2
mGwc5-2	5	299.08	9.03	11.85	73,914,985	83,978,927	16	184	5
mGwc5-3	5	350.96	8.95	22.28	168,316,026	172,575,919	9	71	6
mGwc5-4	5	431.14	7.21	47.85	181,125,890	192,916,785	4	285	4
mGwc5-5	5	510.51	7.54	17.19	206,831,154	208,053,559	4	37	3
mGwc5-6	5	564.78	6.62	4.36	212,658,082	213,160,536	8	8	3
mGwc6-4	6	158.21	6.44	6.08	98,835,925	102,923,288	4	85	4
mGwc6-5	6	312.14	7.88	29.2	148,059,762	150,858,297	4	65	4
mGwc7-3	7	189.99	5.23	0.74	87,807,639	87,955,297	6	1	3
mGwc7-4	7	387.21	11.98	12.17	152,014,115	157,853,917	6	137	4
mGwc8-1	8	45.19	12.77	20.32	3,869,648	5,396,654	2	37	2
mGwc8-2	8	221.9	17.09	15	73,929,318	79,208,663	10	85	7
mGwc8-3	8	269.41	10.32	28.56	96,231,156	103,966,700	8	127	6
mGwc8-4	8	373.24	10.83	20.61	135,010,063	145,349,722	3	245	2
mGwc8-5	8	391.12	16.80	2.35	152,663,921	155,408,748	6	75	3
mGwc9-2	9	205.01	7.90	35.82	22,961,206	35,461,716	4	221	3
mGwc9-3	9	253.19	6.23	43.82	89,184,076	111,488,710	5	364	5
mGwc9-4	9	317.79	6.77	10.2	124,996,394	128,616,100	3	89	2
mGwc10-1	10	167.63	8.16	28.97	10,001,171	17,764,248	6	134	5
mGwc10-2	10	269.14	10.93	14.23	113,709,859	117,886,809	6	86	5

For GWC, in total, 34 MQTLs were formed from the initial QTLs detected in two or more experiments, a reduction of 83% on the number of initial QTL (Fig. 3a, Table 2). The average CI for these MQTLs was 22.13 cM, ranging from 0.74 (mGwc7-3 on linkage group 7) to 91.86 cM (mGwc3-5 on linkage group 3) (Table 2). This is an improvement in resolution of 65% when compared to the average QTL interval for initial QTL (63.67 cM). Similarly, there were significant differences in the mean CI of multiple QTLs among different chromosomes. The mean CI for MQTLs on chromosome 7 was reduced by 91%, but only reduced by 32% on chromosome 9 (Fig. 3c). Compared with 34.1 cM in study result of Xiang et al. [17], the average CI had also been greatly improved. The highest number of MQTLs was 8 which located in linkage group 2, and no MQTLs for GWC were found in linkage group 1.

In order to verify the accuracy of the MQTLs we obtained, we compare our MQTLs results with the research results of Sala et al. [21]. Fourteen overlap-domains were existed between the MQTLs of our obtained and the MQTLs in the study of Sala et al. (Supplementary Table 2). Meanwhile, the accuracy of the these MQTLs we obtained in overlap-domains had been greatly improved. The average CI of these MQTLs we obtained in overlap-domains was 34.04 cM which reduced the average interval 58.48% compared to the average CI for the MQTLs (81.99 cM) that Sala et al. obtained in overlap-domains (Supplementary Table 2). Especially, the mGwc4-2 and S17 were overlapped on 250.2-253.7 cM on chromosome 4. On the basis of S17, the CI of mGwc4-2 was reduced from 118.71 cM to 3.50 cM (Supplementary Table 2). *ZmFIE1* (*Zm00001d049608*) was a gene identified in mGwc4-2. Its homologous gene in Arabidopsis, *FIE1*, could inhibit the expression of the flowering gene *FLC*, thereby affecting plant flowering [25]. Changes in plant flowering can lead to changes in plant growth, which can affect grain moisture content at harvest. In addition, the mGwc6-4 and S22 were overlapped on 155.2-161.3 cM on chromosome 6. Relative to the CI of S22 (32.44 cM), the CI of mGwc6-4 was reduced to 6.08 cM. *ZmTGL1* (*Zm00001d036773*) was a gene identified in mGwc6-4. According to the gene annotation on MaizeGDB (https://www.maizegdb.org/), ZmTGL1 catalyzes lipid degradation in maize. Studies showed that lipid content in maize grains was negatively correlated with GDR [26]. *ZmTGL1* may affect grain dehydration by affecting the metabolic processes in the grain.

### Candidate genes related GDR and GWC identified in MQTL regions

**Fig. 4:**
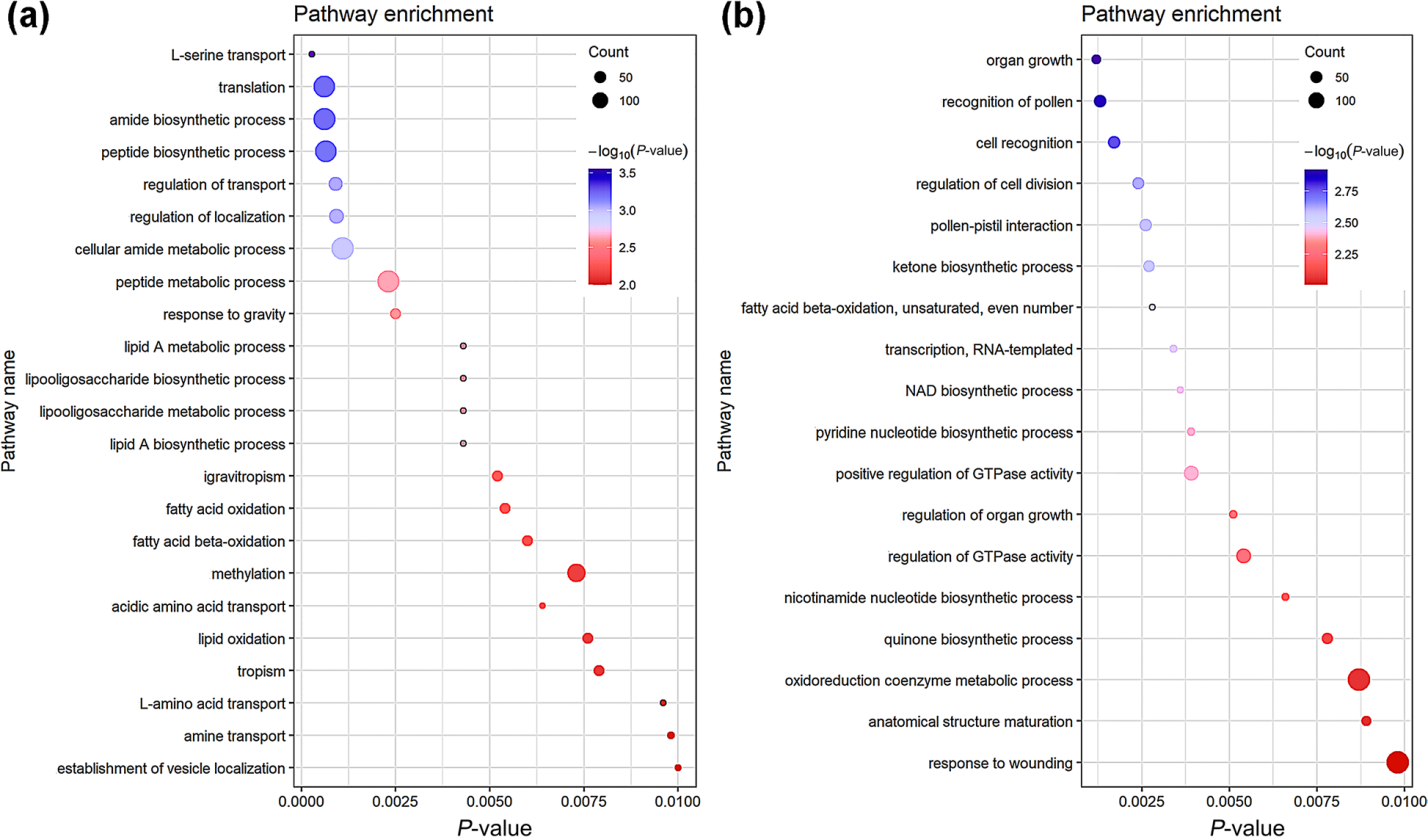
GO enriched in the genes in MQTLs related to GDR (**a**) and GWC (**b**). The parameters are *P*-value ≤0.01

By comparing the positions of the genetic markers at both ends of the MQTL on the B73 genome (AGPv4) [27], a great quantity candidate genes were identified in the selected intervals of MQTLs related GDR and GWC. A total of 1494 candidate genes related to GDR and 5011 candidate genes related to GWC were identified in the current MQTL intervals (Table 2, Supplementary Data 3). We then used GO enrichment analysis to assign candidate genes to functional categories (Fig. 4).

The results for biological processes showed that the candidate genes related GDR were enriched in many biological processes related to lipid homeostasis, such as “lipid A metabolic process”, “lipid A biosynthetic process”, “fatty acid oxidation” and so on (Fig. 4). Lipid homeostasis could affect the transportation of nutrients from the maternal placenta to the developing endosperm, thereby affecting the grain filling process [28]. Studies had shown that there was a certain relationship between the grain filling rate and GDR [5]. Therefore, lipid homeostasis may affect the dehydration process of grains by affecting grain filling. Among all 27 candidate genes related lipid homeostasis, 15 genes were expressed in seed (Supplementary Fig. 2). 7 genes were highly expressed before 10 days after pollination (DAP), 1 gene was highly expressed between 10 DAP and 30 DAP, 2 genes were highly expressed after 30 DAP, and 5 genes were highly expressed before 10 DAP and after 30 DAP.

Genes associated with GWC were also enriched in some biological processes related to lipid metabolism. In addition, many genes were associated with energy metabolism and organization development, such as “Nicotinamide adenine dinucleotide (NAD) biosynthetic process” and “organ growth” (Fig. 4). The grain filling process required respiration to provide energy, and NAD was an important product of respiration. The NAD biosynthetic process may affect the dehydration process by affecting grain filling. Among all 9 candidate genes related NAD biosynthetic process, 8 genes were expressed in seed. 5 genes were highly expressed before 10 DAP, 2 genes were highly expressed before 30 DAP, and 1 gene was highly expressed during all seed developmental process (Supplementary Fig. 3). Among all 16 candidate genes related organ growth, 14 genes were expressed in seed. Seven genes were highly expressed before 10 DAP, 1 gene was highly expressed between 10 DAP and 30 DAP, 3 genes were highly expressed after 30 DAP, and 3 genes were highly expressed before 10 DAP and after 30 DAP (Supplementary Fig. 3). This indicated that GWC may be influenced by the process of early grain development, particularly that of the embryo, which required a lot of energy. This factor had been neglected in previous studies on GWC, and the process of grain morphogenesis prior to the dehydration process should also be paid attention to.

**Fig. 5:**
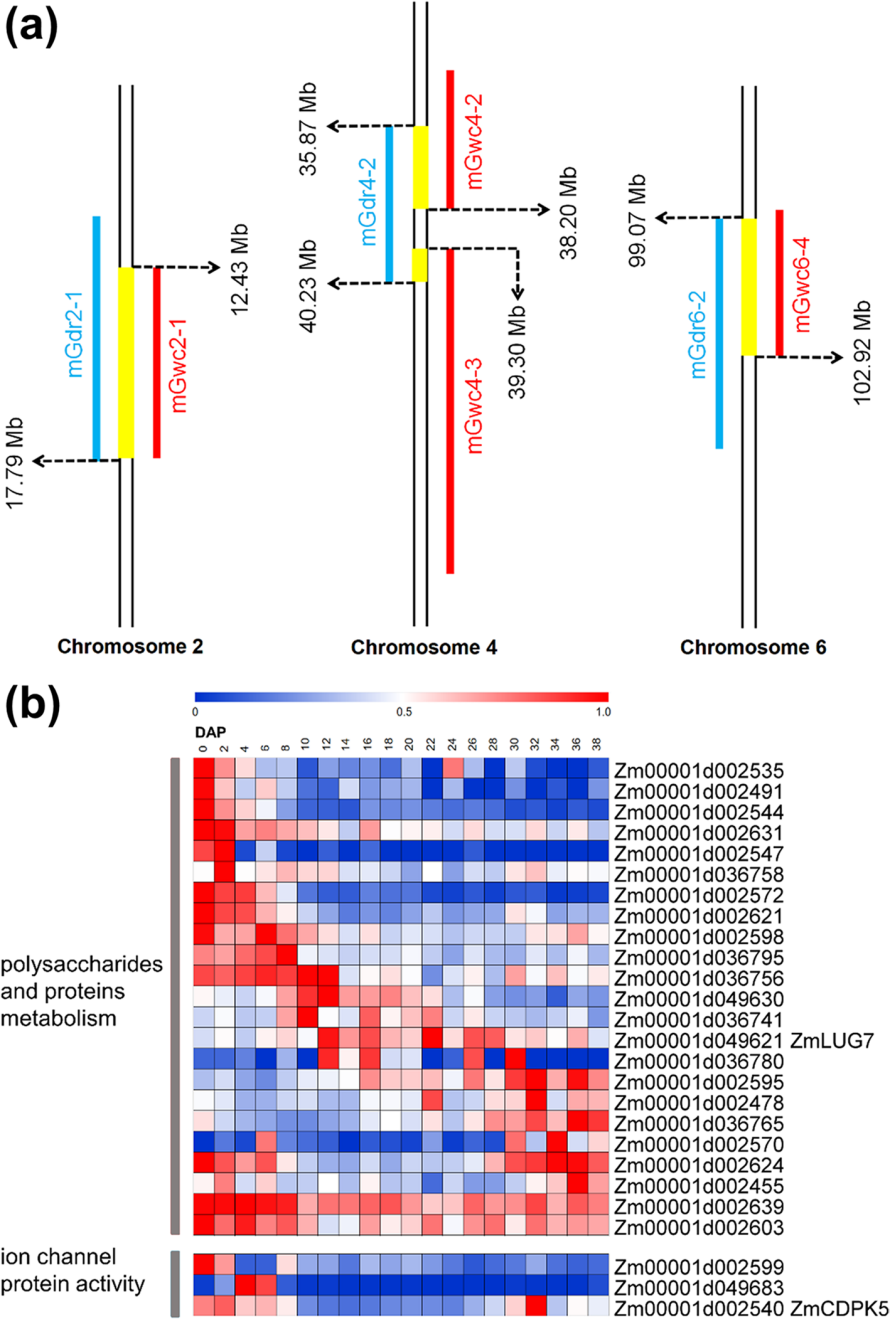
Information of overlap domains between the GDR and GWC-related MQTLs. **a**, chromosome position of the domain on maize genome (AGPv4) which was GDR and GWC-related MQTLs overlap with 95% CI. The MQTLs related to GDR are marked in blue, the MQTLs related to GWC are marked in red, and the overlap domains between the GDR and GWC-related MQTLs are marked in yellow. **b**, expression pattern of partial genes contained in domain where GDR and GWC-related MQTLs overlap in seed during 0-38 DAP

MQTL analysis indicated that some MQTLs associated with GDR and GWC were clustered in the same regions of certain chromosomes (Fig. 5a). A total of 323 overlapping candidate genes were identified on both of GDR and GWC MQTL (Supplementary Data 4). Go enrichment analysis showed that there were many genes related to the degradation of polysaccharides and proteins, and some genes were related to the regulation of ion channel protein activity (Supplementary Fig. 4). There was a layer of waxy cuticle on the surface of maize grain, which may influence the water loss in maize grain to some extent. Among all 45 candidate genes related polysaccharides and proteins metabolism, 23 genes were expressed in seed. Eleven genes were highly expressed before 10 DAP, 4 genes were highly expressed between 10 DAP and 30 DAP, 4 genes were highly expressed after 30 DAP, and 4 genes were highly expressed before 10 DAP and after 30 DAP (Fig. 5b). In addition, it also contained 5 genes related to the regulation of ion channel protein activity.

Comparing these GDR and GWC-related candidate genes with the results of genome-wide association study (GWAS) mapping of GDR and GWC published [4, 29–32], the results showed that there are 7 GDR-related and 14 GWC-related genes in the candidate genes mapped by GWAS (Supplementary Fig. 5, Supplementary Table 3). According to annotation, 5 of these genes were involved in protein degradation, and *Zm00001d007793* in mGdr2-5 and *Zm00001d002535* in mGdr2-1 and mGwc2-1 were involved in protein degradation through the E3 ubiquitin ligase pathway. Previous reports had confirmed that part of E3 ubiquitin ligase may be involved in water transfer in plants [33, 34].

### Analysis of key hormone candidate genes related GDR and GWC

**Fig. 6:**
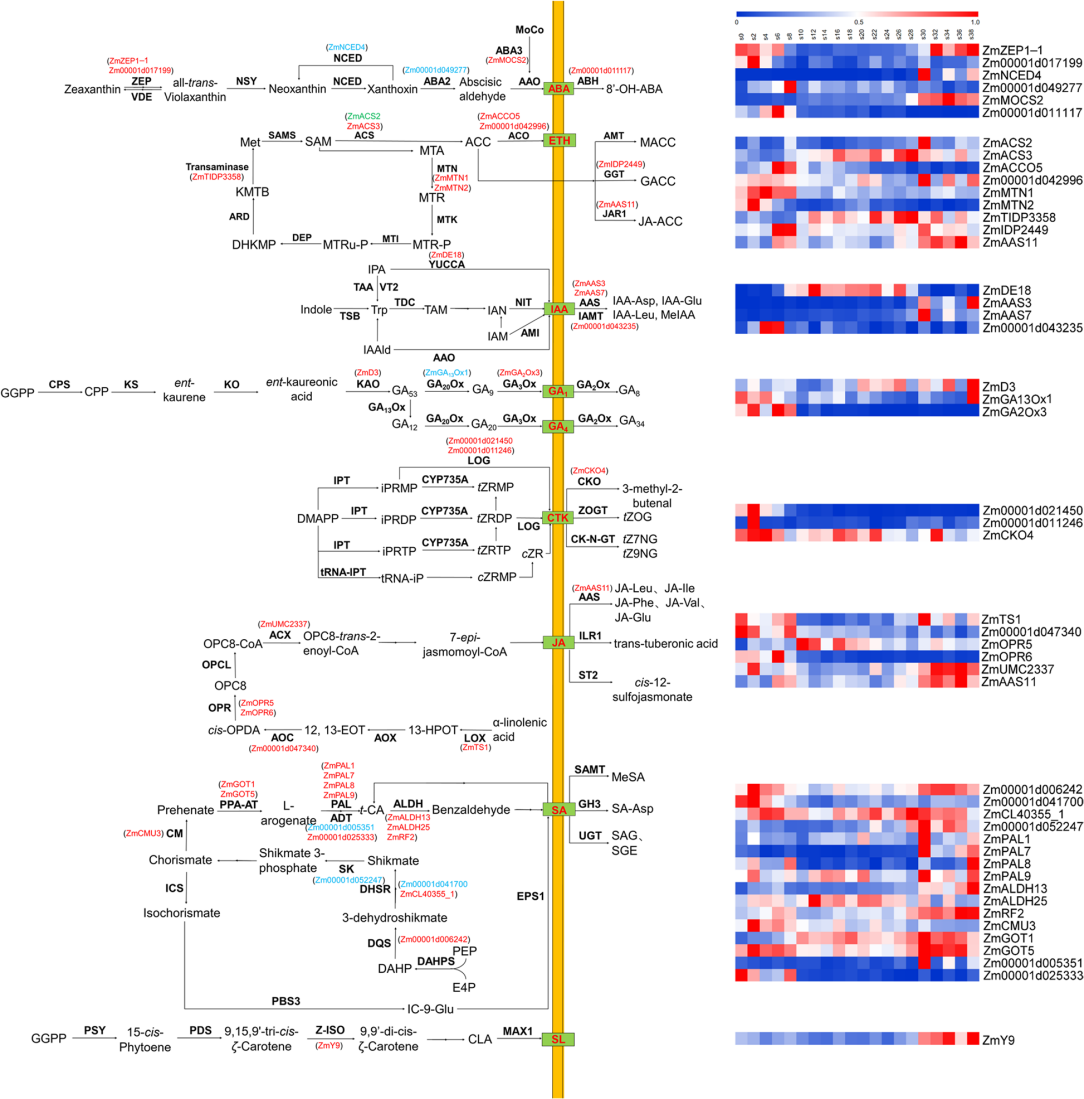
The presence of MQTLs related to GDR and GWC in ABA, ETH, IAA, GA, CTK, JA, SA and SL metabolism pathways. The genes related to GDR were marked in blue, and those related to GWC were marked in red. The green genes represented candidate genes that are related to both GDR and GWC

The growth and development of maize grains was regulated by a variety of plant hormones. In total, 72 genes related to hormones were detected (Supplementary Table 4), and we found 48 of these were expressed in seed [35, 36], including 6 genes related ABA, 9 gene related ETH, 4 genes related IAA, 3 genes related GA, 3 genes related cytokinin (CTK), 6 genes related jasmonic acid (JA), 16 genes related salicylic acid (SA) and 1 gene related strigolactone (SL) (Fig. 6).

ABA had been shown to increase GDR during grain development [37]. We found 2 GDR-related candidate genes and 4 GWC-related candidate genes to ABA metabolism (Fig. 6). *ZmMOCS2* (*Zm00001d006342*) was identified in mGwc2-5, and encoded a molybdenum cofactor sulfurase (MoCo). *ZmMOCS2* were expressed highly after 30 DAP (Fig. 6). *LOS5*, a gene encoded MoCo in Arabidopsis, could regulates the last step of ABA biosynthesis [38]. Previous report showed that overexpressed *LOS5* in maize could significantly increase the activity of aldehyde oxidase, which resulted in the accumulation of ABA and improved the drought resistance of maize [38]. The zeaxanthin epoxidase (ZEP) encoded by *ZmZEP1* (*Zm00001d003512*) in mGwc2-2 and *Zm00001d017199* in mGwc5-4, 9-cis-epoxycarotenoid dioxygenase (NCED) encoded by *ZmNCED4* (*Zm00001d007876*) in mGdr2-5, xanthoxin dehydrogenase (ABA2) encoded by *Zm00001d049277* in mGdr4-1 and abscisic acid 8′-hydroxylase (ABH) encoded by *Zm00001d011117* in mGwc8-4 were key enzymes in the process of ABA synthesis and degradation [39]. *ZmZEP1* was highly expressed before 6 DAP and after 32 DAP (Fig. 6). *ZmZEP5, Zm00001d049277* and *Zm00001d011117* were expressed highly before 8 DAP, while *ZmNCED4* was highly expressed after 30 DAP (Fig. 6). This showed that ABA in the process of grain dehydration was likely to be degraded mainly through 8′-hydroxylase pathways.

ETH was a key regulator of starch synthesis and PCD during plant development [6]. We found 1 GDR-related candidate genes and 9 GWC-related candidate genes to ETH metabolism (Fig. 6). *ZmACS2* (*Zm00001d002592*) was a gene present in both mGdr2-1 and Gwc2-1. *ZmACS2* was highly expressed after 30 DAP (Fig. 6). *ZmACS2* and *ZmACS3* (*Zm00001d045479*) in mGwc9-2 encoded a ACC synthase (ACS). *ZmACCO5* (*Zm00001d011208*) in mGwc8-4 and *Zm00001d042996* in mGwc3-5 encoded a ACC oxidase (ACO). ACS and ACO were all key enzymes in the process of ethylene synthesis [40]. *ZmACS3* was highly expressed after 14 DAP, *ZmACCO5* was highly expressed before 8 DAP and *Zm00001d042996* was highly expressed before 6 DAP and after 30 DAP (Fig. 6). Moreover, the 5′-methylthioadenosine nuclease (MTN) encoded by *ZmMTN1* (*Zm00001d049823*) in mGwc4-3 and *ZmMTN2* (*Zm00001d049823*) in mGwc9-2, transaminase encoded by *ZmTIDP3358* (*Zm00001d013992*) in mGwc5-1 were both enzymes in the synthesis of ETH. *ZmMTN1* and *ZmMTN2* were highly expressed before 8 DAP, and *ZmTIDP3358* was highly expressed after 12 DAP (Fig. 6). The glutamyltransferase (GGT) encoded by *ZmIDP2449* (*Zm00001d003446*) in mGwc2-2 and jasmonate resistant 1 (JAR1) encoded by *ZmAAS11* (*Zm00001d009714*) in mGwc8-2 could inactivate ACC, the direct precursor of ETH synthesis [41]. Both *ZmIDP2449* and *ZmAAS11* were mainly expressed before 8 DAP and after 30 DAP (Fig. 6). For *ZmAAS11*, except for ETH, it also plays an important role in the metabolic pathways of JA which indicates that these hormones may act together in the dehydration process of grains [42].

IAA could regulate plenty of aspects of plant growth and development. Recent studies showed that IAA could promote the expression of starch biosynthesis genes in rice [43]. In addition, IAA could promote plant resistance to environmental stress by controlling the expression of *Gretchen Hagen 3* (*GH3*) and other IAA-responsive family genes [44]. We found 4 GWC-related candidate genes to IAA metabolism (Fig. 6). *ZmAas3* (*Zm00001d007395*) identified in mGdr2-7 and *ZmAas7* (*Zm00001d043350*) identified in mGwc3-5 were genes of the GH3 family. *ZmAas3* and *ZmAas7* were highly expressed after 30 DAP (Fig. 6). The expression of *ZmAas3* in shoots could be induced by sodium chloride treatment, which may be related to the response of maize to water deficit stress [45]. *Zm00001d043235* in mGwc3-5 was a gene related IAA decomposition, which was highly expressed before 6 DAP (Fig. 6). *ZmDE18* (*Zm00001d023718*) in mGwc10-1 was a gene in the synthesis of IAA [46], which was highly expressed between 8 DAP and 28 DAP (Fig. 6).

GA was an important type of plant hormone, which played an important role in the process of plant flowering induction and fruit development [47, 48]. We found 1 GDR-related and 2 GWC-related candidate genes to GA biosynthesis [49] (Fig. 6). The ent-kaurenoic acid oxidase (KAO) encoded by *ZmD3* (*Zm00001d045563*) in Gwc9-2, GA_13_oxidase (GA_13_Ox) encoded by *ZmGA*_*13*_*Ox1* (*Zm00001d007180*) in mGdr2-4 and GA_2_oxidase (GA_2_Ox) encoded by *ZmGA*_*2*_*Ox3* (*Zm00001d043411*) in mGdr3-5. *ZmD3* was highly expressed after 20 DAP, *ZmGA*_*13*_*Ox1* was highly expressed before 4 DAP and after 38 DAP (Fig. 6). *ZmGA*_*2*_*Ox3* was highly expressed before 8 DAP (Fig. 6).

CTK plays a prominent role in promoting cell division, and the CTK in maize grains mainly occurs in the endosperm, and the concentration of CTK in the developing embryo is very low. Therefore, it is speculated that CTK promotes cell division in the endosperm [50]. We found 3 GWC-related candidate genes to CTK metabolism (Fig. 6). Cytokinin-activating enzyme encoded by *Zm00001d021450* in mGwc7-4 and *Zm00001d011246* in mGwc8-4 was a key enzyme in the synthesis of CTK [51]. The expression levels of these two genes were highly before 4 DAP (Fig. 6). The CTK oxidase (CKO) encoded by *ZmCKO4* (*Zm00001d043293*) in mGwc3-5 could catalyze the degradation of CTK [51], which was highly expressed in the whole grain development stage (Fig. 6).

JA and SA mainly played a role in the defense of plant diseases and insect pests, and few studies had been done on their role in the development of maize grains. It was currently known that SA may antagonize each other with IAA [52] and affected the formation of sugar in maize grains [53]. For JA, 6 genes related GWC were detected (Fig. 6). Of them, the expression of *Zm00001d047340* in mGwc9-4 was significantly higher than that of other genes. It encoded an allene oxide cyclase (AOC) and was highly expressed before 8 DAP (Fig. 6). Furthermore, the 16 SA-related candidate genes we detected were all genes related to SA synthesis, and no expressed SA degradation-related genes were detected [54] (Fig. 6). Among them, *ZmGOT1* (*Zm00001d043382*) in mGwc3-5 had the highest expression level, and the expression levels of other SA-related genes were 30 to 40 times higher. Its expression level was high after 10 DAP.

SL played an important role in regulating flowering time of plants, and could regulate flowering time of plants together with melatonin [55]. Previous studies had shown that there was a “safe threshold” for melatonin regulation of flowering in Arabidopsis, within which SL inhibits flowering by inhibiting the expression of *SPL* gene. Outside the threshold range, strigolactone inhibits flowering by inhibiting melatonin synthesis, which in turn promotes the expression of the flowering suppressor gene *FL*C [55]. *ZmY9* (*Zm00001d023655*) in mGwc10-1 encoded a 15-cis-ζ-carotene isomerase (Z-ISO) and played a role in the biosynthesis of SL [56]. It was highly expressed after 30 DAP (Fig. 6).

## Discussion

Maize is one of the most important crops in the world and an important source of feed, food, energy and industrial raw materials. However, the excessively high GWC during maize harvest in my country severely limits the efficiency of mechanized harvesting and increases the cost of drying and storage [57]. Therefore, breeders regard GDR and GWC at harvest as one of the important indicators of molecular marker-assisted breeding.

### Genetic architecture of GDR and GWC revealed by MQTL analysis

To gain a better understanding of the genetic architecture of GDR and GWC, we performed MQTL analysis using reported QTL from previous mapping experiments. In total, 282 QTLs from 25 previous QTL mapping experiments were projected onto the integrated linkage map successfully (Table 1, Supplementary Data 2).

Since the 1990s, there had been many experiments on QTL mapping related to maize grain moisture [12, 15, 22, 24]. These QTL mapping experiments could be divided into two categories: QTL mapping related to GDR and GWC. However, the number of experiments and QTLs related to GWC was far more than them related to GDR. Reasons may be that GDR was more susceptible to environmental conditions than GWC. Undoubtedly, the effectiveness of marker—assisted selection strongly depended on the accuracy of QTL mapping results in terms of position and effects of the detected QTL. Analysis of quantitative trait relationships is important to evaluate the feasibility of joint selection of two or more traits [58, 59]. Previous studies have shown that GDR is a key determinant of GWC [3]. Here, we performed co-localization analysis of GDR and GWC-related MQTLs and found that there were only 3 overlapping regions containing 323 candidate genes. Col-localization of MQTLs for the GDR and GWC is in line with the results of previous studies, showing that the variability in GDR and GWC of maize may be due to distinct genetic architectures [4]. However, many of the 323 genes could affect GWC at harvest in various ways. Therefore, in the process of QTL mapping and marker—assisted selection, we could use the GWC at harvest as an indicator and GDR as a reference to improve the effectiveness of marker—assisted selection to a certain extent [60]. There are many co-localized genes between GDR and GWC, suggesting that there may be some common regulatory mechanisms between these two traits.

The interaction between different QTLs had a greater impact on GWC than a single QTL. Among the results of QTL mapping for GWC, the likelihood of odd (LOD) value of the final QTL was mostly high, but the explanatory variation rate of the phenotype was mostly very low. The maximum explanatory variation rate of the phenotype in many articles was below 10%. Combined with the existing research results, there were significant interactions between QTL and QTL (more effective in the early stage of grain dehydration), and between QTL and the environment (more effective in the late stage of grain dehydration) [4]. These two interactions may have a more significant impact on grain moisture than a single QTL. This indicated that it may be a more effective method to study the dehydration process of seeds from a biological process rather than from genes.

Previous studies had shown that the grain dehydration process may be affected by biological processes such as starch synthesis and PCD [10]. Hormones played an important regulatory role in these two biological processes. ABA and ETH could antagonize and regulate the PCD process of endosperm cells, and increasing the ratio of ABA to ETH had also been shown to improve the grain filling process of rice. The increase in ABA content had been shown to increase GDR and reduce GWC at harvest. Therefore, it was a good choice to study the grain dehydration process from the perspective of hormones. Among the candidate genes in the MQTLs we obtained, 48 genes related to hormone metabolism were detected (Fig. 6). These genes and corresponding hormones had complex interactions in the process of grain development. Studying these 48 genes related to hormone metabolism could help us better understand the process of grain dehydration.

### Other influencing factors of GDR and GWC

The dehydration process of grains was affected by many factors [5]. In addition to the starch synthesis and PCD that had been reported before, it was also affected by many other factors. The results of GO enrichment analysis contained many biological processes related to lipid homeostasis, “lipid A metabolic process”, “lipid A biosynthetic process”, “fatty acid oxidation” and so on (Fig. 4), indicating that lipid homeostasis may also have a certain impact on the dehydration process of grains. The large enrichment of genes related to energy metabolism and organization development in GWC-related genes. There were some genes related to early embryo development of grains contained in GWC-related genes, which indicated that the early development of grain, which had been neglected in many previous studies, may also affect grain dehydration. Many genes related to energy metabolism were enriched, which was consistent with the high energy requirement during grain development.

## Conclusions

In this study, MQTL analysis was performed using 282 QTLs from 25 studies to propose MQTL on a high-density genetic linkage map. For GDR and GWC, a total of 11 and 34 MQTLs were identified. The MQTLs identified possessed a narrower CI than the original QTLs. with the CI of MQTLs related to GDR and GWC were 24.44 and 22.13 cM, respectively. A total of 1494 candidate genes related to GDR and 5011 candidate genes related to GWC were identified in MQTL intervals. Among these genes, there are 48 genes related to hormone metabolism, indicating that hormones may play an important role in the process of grain dehydration. Of these 48 genes, 45 genes are highly expressed across before 8 DAP or after 30 DAP in grain and many genes are related to the starch synthesis and PCD. In addition, factors such as lipid homeostasis, energy metabolism and organization development may also have a certain impact on the process of grain dehydration. Our results provide new ideas for studying grain dehydration, analyzing major locus for regulating GWC in maize, which will help maize breeding.

## Methods

### Collection of QTL information related GDR and GWC of maize

All data were collected from 25 experiments on QTLs related GDR and GWC of maize published from 1992 to 2020 (Table 1). Here, we defined that an experiment was as the QTL analysis of one population for a given trait in a given environment. Collected information related to QTL mapping related GDR and GWC of maize in the literatures, including name, size, type and mapping function of mapping population, trait information, name of QTL, location of chromosome, LOD value, phenotypic contribution rate, additive effect and heredity number and location of map markers.

### QTL projection and MQTL analysis

Projected the identified QTL onto a reference map for MQTL analysis. The projection was performed using BioMercator (v4.2) [61]. For the experiments with complete genetic map and QTL information, the information was arranged according to the format required by the software. For the experiments that did not give the LOD value, the LOD value was uniformly taken as 2.5 [17]; if only CI (95%) or *R*^2^ value of a QTL was known, it could be inferred based on the following formulas:

1 $$\mathrm{CI}=530\div \left(\mathrm{N}\times {R}^2\right)$$

2 $$\mathrm{CI}=163\div \left(\mathrm{N}\times {R}^2\right)$$

In the two formulas, “CI” was the confidence interval of QTL; “N” represented the size of the mapping population; “*R*^*2*^” represented the genetic contribution rate of QTL; formula (1) applied to backcross and F_2_ mapping populations [62]; formula (2) applied to recombinant inbred lines mapping populations [63].

For experiments without genetic map but with markers at both ends of QTLs, put the QTLs of it compare to the reference genetic map IBM2 2008 Neighbors (https://www.maizegdb.org/data_center/map). QTLs that could not be compared with the reference genetic map at both ends of the marker was discarded directly. For QTLs that only one segment of marker can be compared to the reference genetic map successfully, after the CI was calculated according to the above formula, the genetic position of the marker on the other end can be calculated according to the genetic position of the marker on the comparison. In the experiments of QTL mapping with SNP markers, SNP markers could not be associated with other genetic maps, so MQTL analysis cannot be performed directly. However, the physical location information of QTL obtained by SNP markers was given. Therefore, I manually converted the physical locations at both ends of QTLs to the corresponding genetic locations on the reference genetic map IBM2 2008 Neighbors, and then conducted MQTL analysis.

The MQTL analysis of GDR and GWC were performed separately. All information related to a single genetic map used in this experiment was collected based on published maps. Take the IBM2 2008 Neighbors high-density genetic linkage map as the reference map. The detailed projection program was executed according to the method of Chardon et al. described [64]. We integrated the genetic maps of literatures and the IBM2 2008 Neighbors reference genetic map to get the consensus map. In a few cases, the display order of common markers was inconsistent (the order between maps). Whenever the removal of the marker would not degrade the construction of the consensus graph, it would be discarded from the analysis. The QTL in their original map was projected onto the consensus map, using markers shared by the two maps through similar functions. According to the location and CI of each QTL, the software tested the akaike type criteria (AIC) values of the AIC model when the number of MQTLs on a specific chromosome was 1-10. When selecting the number of MQTL on each chromosome, the number of MQTL corresponding to AIC minimum was considered first. Then performed meta-analysis again to synthesize MQTL analysis.

In order to facilitate the display of the initial QTLs, the genetic map used in Fig. 2 was a genetic map obtained by integrating the GDR and GWC-related integrated genetic maps using IBM2 2008 Neighbors as the reference genetic map by BioMercator (v4.2).

### Acquisition of candidate gene and GO enrichment analysis

After obtaining MQTLs given by the software, MQTLs obtained from at least two experiments were screened out. The physical location of partial markers on B73 (AGPv4) of IBM2 2008 Neighbors could be found on MaizeGDB (https://www.maizegdb.org/) [27], and the physical location of genetic markers closest to both ends of MQTLs was first checked on IBM2 2008 Neighbors. Finally, the precise physical positions at both ends of MQTLs could be obtained by using the following formula [65].

$$\mathrm{p}=\mathrm{p}1+\left(\mathrm{p}2-\mathrm{p}1\right)\times \left(\mathrm{g}-\mathrm{g}1\right)\div \left(\mathrm{g}2-\mathrm{g}1\right)$$

“*g1*” and “*g2*” were the genetic locations of bilateral genetic markers, “*g*” was the genetic location of target genetic markers, “*p1*” and “*p2*” were the physical locations of bilateral genetic markers, and “*p*” was the physical location of target genetic markers.

Finally used the “qTeller” module on MaizeGDB to look up the genes contained in MQTLs as candidate genes.

We used the Agrigo (v2.0) website (http://systemsbiology.cau.edu.cn/agriGOv2/) to perform GO enrichment analysis on candidate genes related GDR and GWC, respectively. When the *P*-value of the term was less than 0.01, we considered it enriched in the corresponding gene set. For the display of GO enrichment analysis results, we used the form of “bubble chart” to display. The drawing of “bubble chart” was done using the R language package (ggplot2) [66].

### RNA-seq data processing and drawing of the chart

For the RNA-seq of maize grains downloaded from the NCBI from 0 to 38 days after pollination [35, 36], we now divided the expression levels at different time points by the maximum observed fragments per kilobase of transcript per million mapped reads (FPKM) to calculate the normalized expression value of the gene. Then used MeV (v4.9) software to draw a heat map of gene expression. The GDR and GWC of the RNA-seq samples was not obtained [35]. According to previous results, the variation trend of GWC of different hybrids that planted at the close planting place and obtained at the similar developmental stage with the RNA-seq samples, was showing a “S”-shaped downward trend of “slow-fast-slow” [67, 68] and the GDR showed a unimodal curve that first increased and then decreased with the increase of silking days [68].

## Supplementary Information

**Additional file 1.**

## Data Availability

All data supporting the conclusion of this article are provided within the article and its supplementary.

## Abbreviations

ABA: Abscisic acid
ABH: Abscisic acid 8′-hydroxylase
ACO: ACC oxidase
ACS: ACC synthase
AIC: Akaike type criteria
AOC: Allene oxide cyclase
AVG: 2-aminoethoxyvinylglycine
CI: Confidence interval
CKO: CTK oxidase
CTK: Cytokinin
DAP: Days after pollination
ETH: Ethylene
FPKM: Fragments per kilobase of transcript per million mapped reads
GA: Gibberellin
GA_2_Ox: GA_2_oxidase
GA_13_Ox: GA_13_oxidase
GDR: Grain drying rate
GGT: Glutamyltransferase
GH3: Gretchen Hagen 3
GO: Gene ontology
GWAS: Genome-wide association study
GWC: Grain water content
IAA: Auxin
JA: Jasmonic acid
JAR1: Jasmonate resistant 1
KAO: ent-kaurenoic acid oxidase
LOD: Likelihood of odd
MoCo: Molybdenum cofactor sulfurase
MTN: 5′-methylthioadenosine nuclease
MQTL: Meta-QTL
NAD: Nicotinamide adenine dinucleotide
NCED: 9-cis-epoxycarotenoid dioxygenase
PCD: Programmed cell death
PVE: Phenotypic variation explained
QTL: Quantitative trait locus
RIL: Recombinant inbred line
SA: Salicylic acid
SL: Strigolactone
ZEP: Zeaxanthin epoxidase
Z-ISO: 15-cis-ζ-carotene isomerase
