# Supplementary material for: Meta-QTL analysis explores the key genes, especially hormone related genes, involved in the regulation of grain water content and grain dehydration rate in maize
Source: BMC Plant Biol. 2022 Jul 16;22:346. doi: 10.1186/s12870-022-03738-y (PMC9287936; doi:10.1186/s12870-022-03738-y)
Supplement: Supplementary file 1 — Additional file 1. [file 12870_2022_3738_MOESM1_ESM.zip › Additional files/Supplementary Figure 1-5 and Table 1-4.v6.docx]

**Meta-QTL analysis explores the key genes, especially hormone related genes, involved in the regulation of grain water content and grain dehydration rate in maize**

Wei Wang^1^, Zhaobin Ren^1^, Lu Li^1^, Yiping Du^1^, Yuyi Zhou^1^, Mingcai Zhang^1^, Zhaohu Li^1^, Fei Yi^1^*, Liusheng Duan^1, 2^

^1^State Key Laboratory of Plant Physiology and Biochemistry, Engineering Research Center of Plant Growth Regulator, Ministry of Education &College of Agronomy and Biotechnology, China Agricultural University, No.2 Yuanmingyuan West Road, Haidian, Beijing 100193, China

^2^College of Plant Science and Technology, Beijing University of Agriculture, No.7 Beinong Road, Changping, Beijing 102206, China

*** Corresponding author：**

Fei Yi

Engineering Research Center of Plant Growth Regulator, China Agricultural University, Beijing 100193, P.R. China

Email: yifei56@cau.edu.cn

**Key words:** Grain water content, Grain dehydration rate, QTL, Meta-analysis, Hormone

**Supplementary Information**

**Additional file 1: Supplementary Fig. 1** Information on initial QTLs. a, chromosome-wise distribution of QTLs; b, confidence interval of the QTLs (used for projection); c, frequency distribution for the number of phenotypic variation explained of the QTLs.

**Additional file 2: Supplementary Fig. 2** Expression pattern of lipid homeostasis genes related GDR in 0-38 DAP.

**Additional file 3: Supplementary Fig. 3** Expression pattern of NAD biosynthetic process and organ growth genes related GWC in 0-38 DAP.

**Additional file 4: Supplementary Fig. 4** GO enriched in the genes in overlap-domain between the MQTLs related to GDR and GWC. The parameters are *P*-value ≤ 0.01.

**Additional file 5: Supplementary Fig. 5** Genomic collinearity of the MQTLs in the GWAS results for GDR and GWC. The MQTLs are shown on the right side of each chromosome. The genomic positions of the MQTL regions correspond to Table 2. Distances on the map are in Mb. The MQTLs related to GDR are marked in blue, and those related to GWC are marked in red. The genes identified by GWAS for GDR and GWC are displayed on the left side of each chromosome (Supplementary Table 3).

**Additional file 6: Supplementary Table 1** Information of GDR and GWC MQTLs we got through MQTL analysis.

**Additional file 7: Supplementary Table 2** Comparison of previous and our research results.

**Additional file 8: Supplementary Table 3** Comparison of GWAS and our research results.

**Additional file 9: Supplementary Table 4** Candidate genes related to hormones.

**Additional file 10: Supplementary Data 1** The information of consensus linkage maps related GDR and GWC.

**Additional file 11: Supplementary Data 2** The information of QTLs mapping successfully related GDR and GWC.

**Additional file 12: Supplementary Data 3** Candidate genes related GDR and GWC identified in MQTL regions.

**Additional file 13: Supplementary Data 4** Genes co-localized with MQTLs related to GDR and GWC.

**Supplementary Figures:**

**
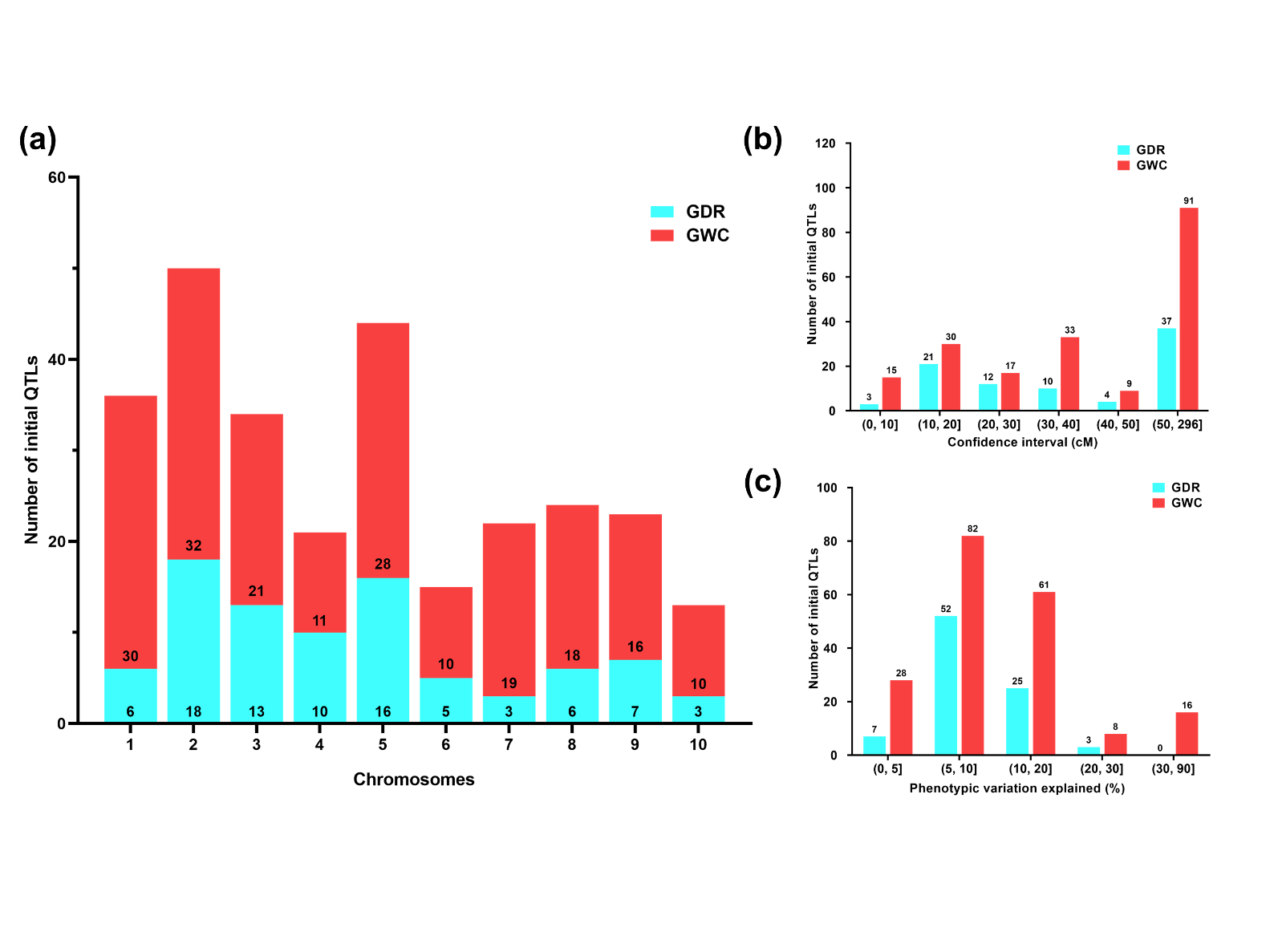
**

**Supplementary Fig. 1** Information on initial QTLs. a, chromosome-wise distribution of QTLs; b, confidence interval of the QTLs (used for projection); c, frequency distribution for the number of phenotypic variation explained of the QTLs.


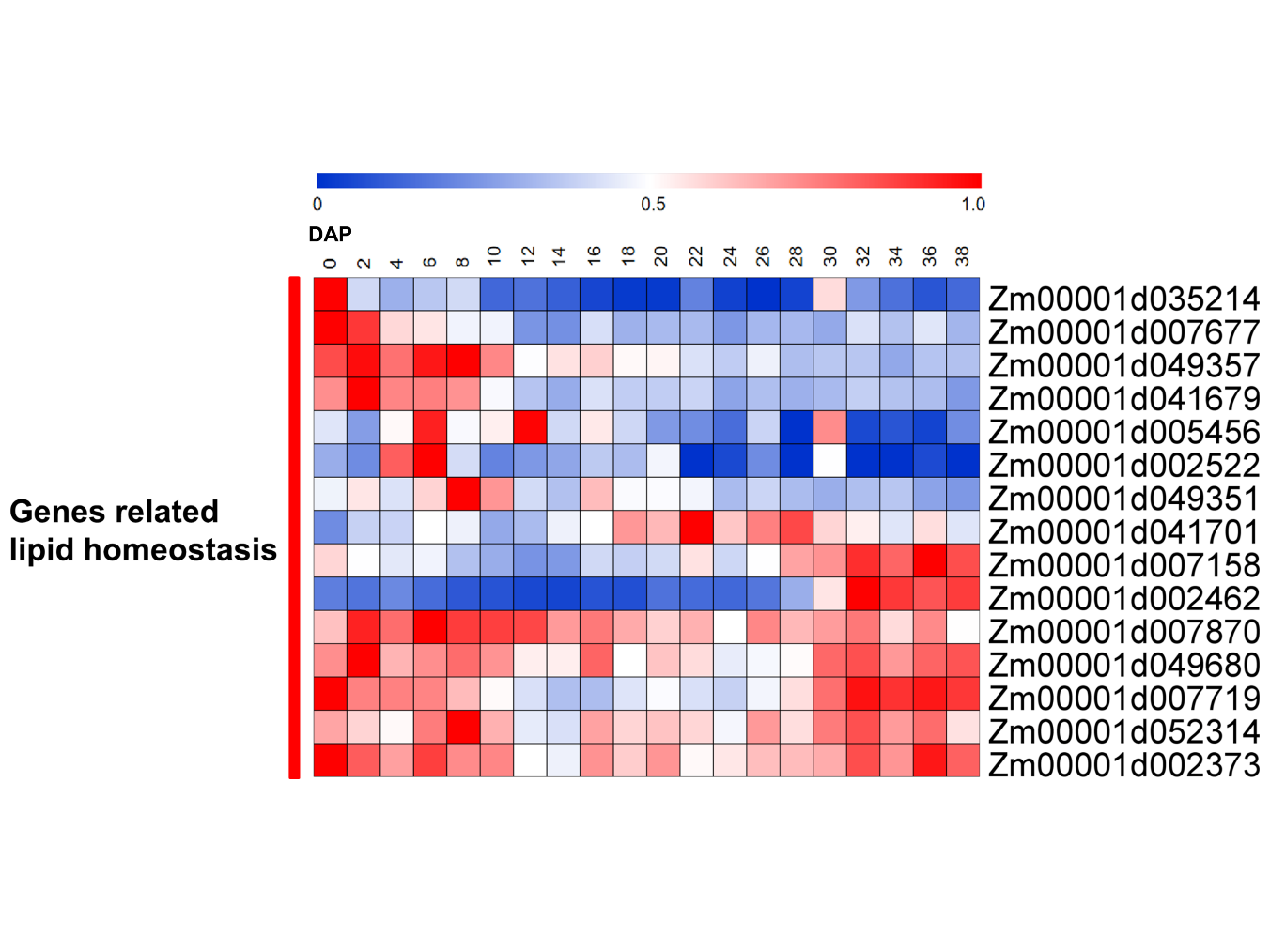


**Supplementary Fig. 2** Expression pattern of lipid homeostasis genes related GDR in 0-38 DAP.


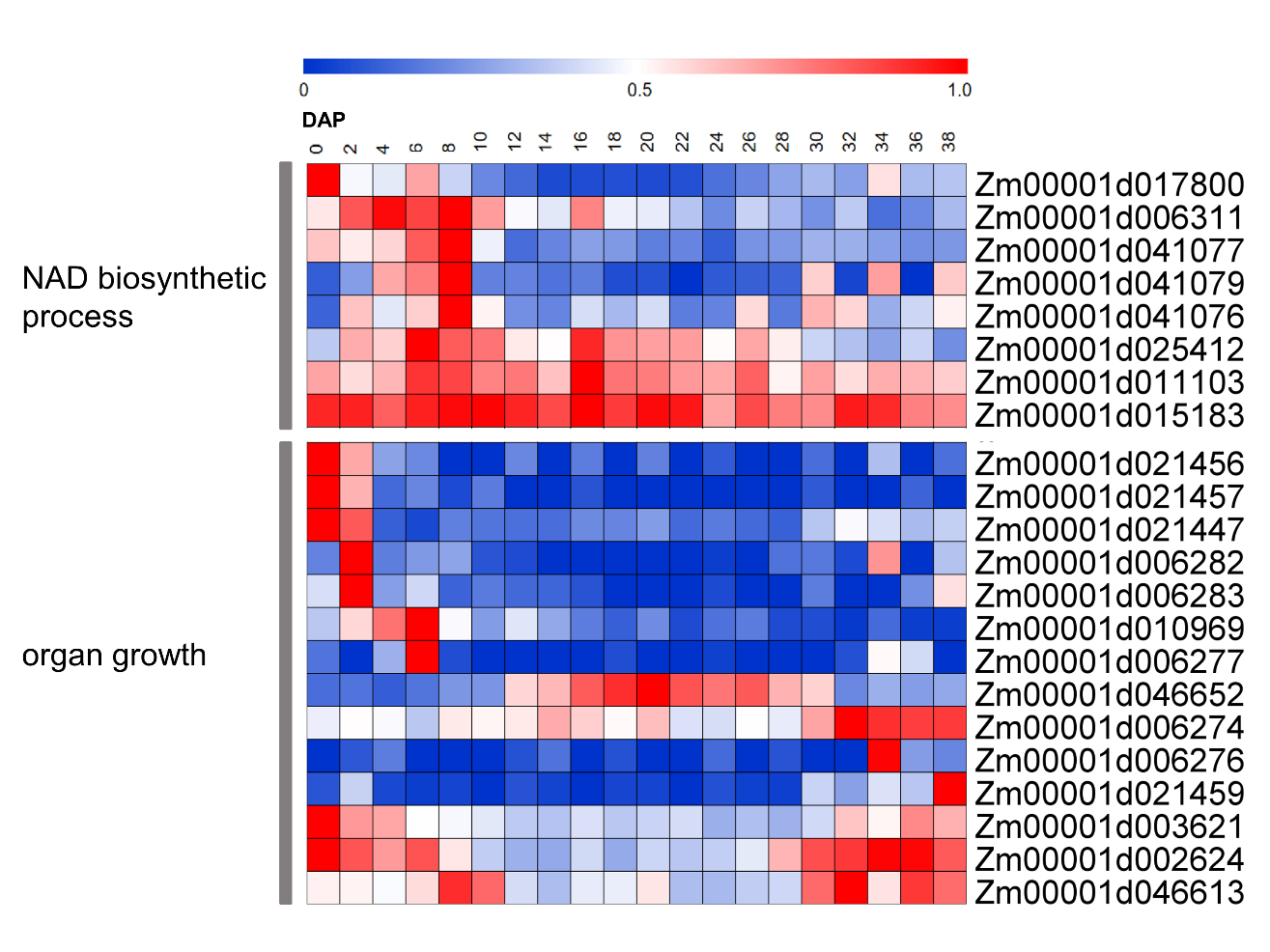


**Supplementary Fig.** **3** Expression pattern of NAD biosynthetic process and organ growth genes related GWC in 0-38 DAP.


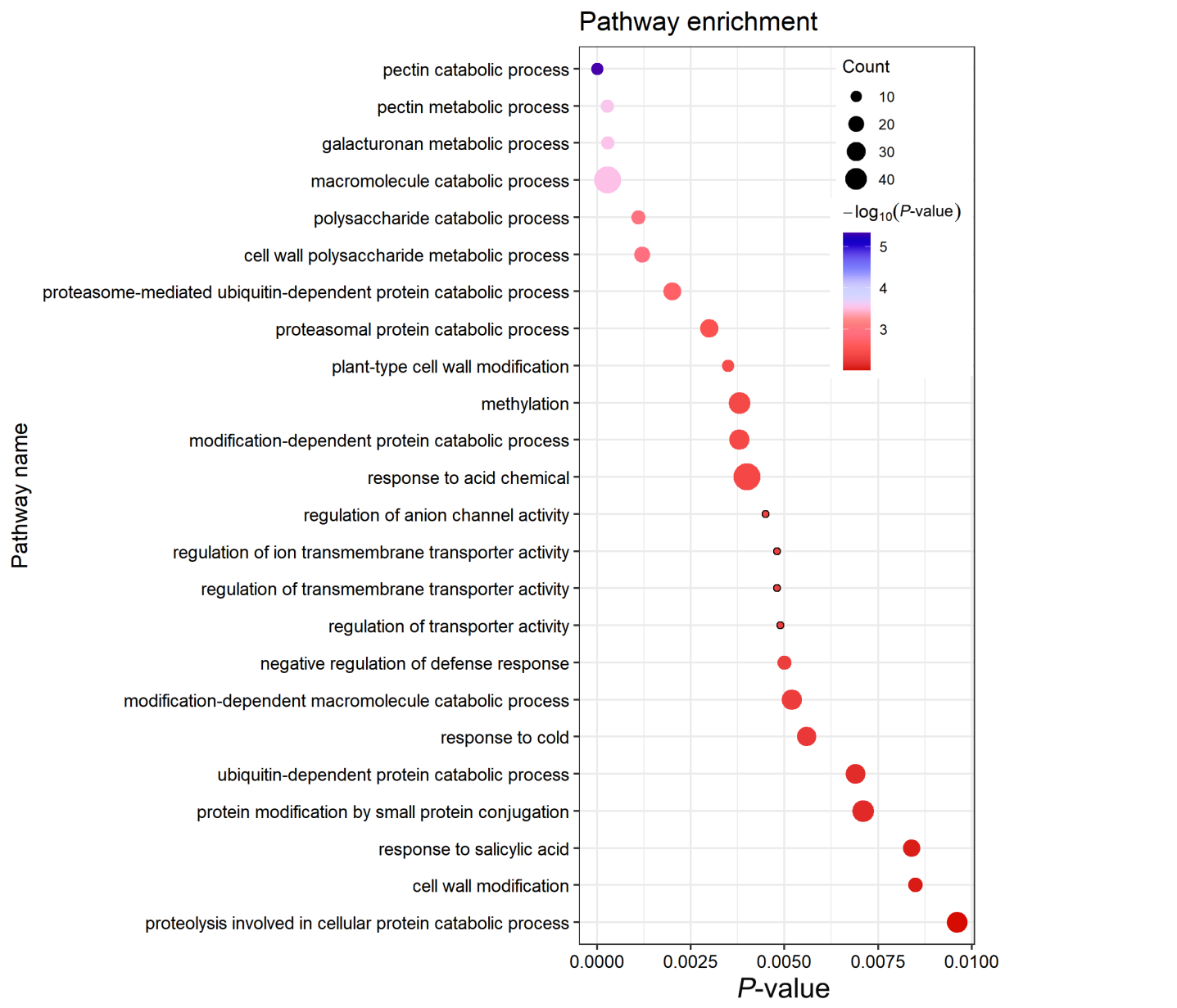


**Supplementary Fig. 4** GO enriched in the genes in overlap-domain between the MQTLs related to GDR and GWC. The parameters are *P*-value ≤ 0.01.


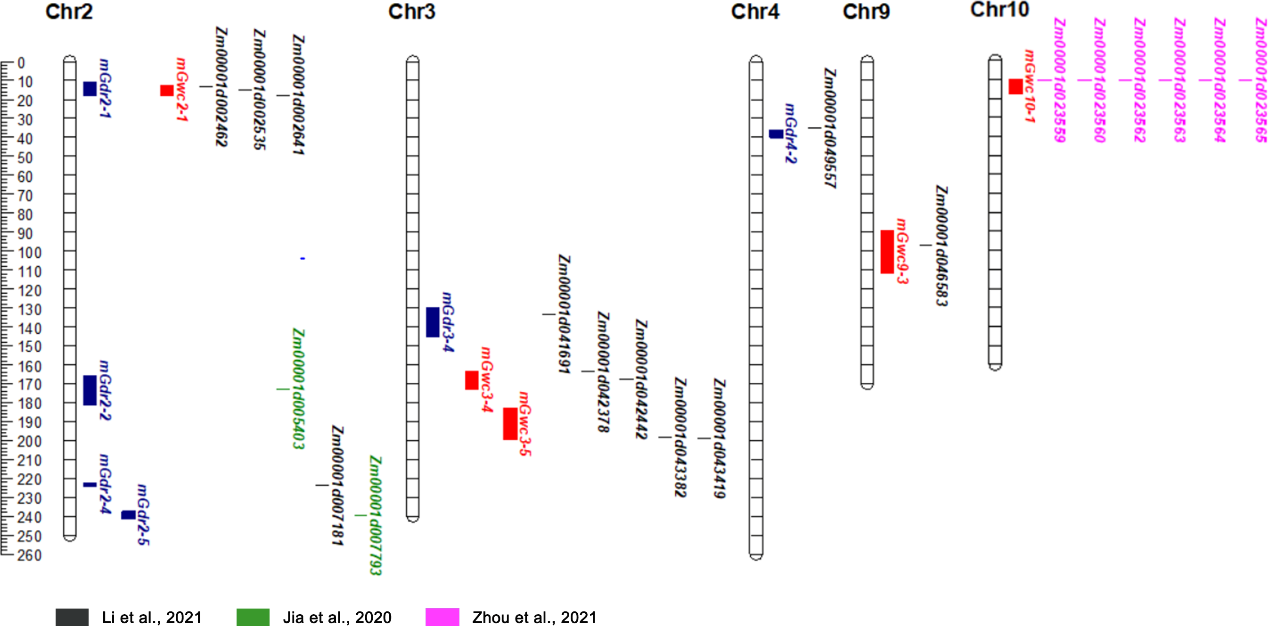


**Supplementary Fig. 5** Genomic collinearity of the MQTLs in the GWAS results for GDR and GWC. The MQTLs are shown on the right side of each chromosome. The genomic positions of the MQTL regions correspond to Table 2. Distances on the map are in Mb. The MQTLs related to GDR are marked in blue, and those related to GWC are marked in red. The genes identified by GWAS for GDR and GWC are displayed on the left side of each chromosome (Supplementary Table 3).

**Supplementary Tables:**

**Supplementary Table 1** Information of GDR and GWC MQTLs we got through MQTL analysis.

| **MQTL** | **Chr.** | **Position (cM)** | **CI (cM)** | **Left Physical Position (bp)** | **Right Physical Position (bp)** | **QTL Intergrated** | **NO. of Gene** | **NO. of studies involved.** |
| --- | --- | --- | --- | --- | --- | --- | --- | --- |
| mGdr3-1 | 3 | 191.07 | 36.94 | 19289020 | 35218326 | 1 | 256 | 1 |
| mGdr3-2 | 3 | 258.62 | 14.18 | 90325459 | 107867255 | 2 | 133 | 1 |
| mGdr3-3 | 3 | 284.64 | 18.77 | 114662187 | 125848733 | 1 | 97 | 1 |
| mGdr3-5 | 3 | 388.15 | 7.8 | 170341045 | 171948645 | 2 | 31 | 1 |
| mGdr4-3 | 4 | 293.64 | 38.05 | 47922505 | 145036483 | 2 | 834 | 1 |
| mGdr9-1 | 9 | 251.17 | 20.17 | 94264587 | 106137193 | 3 | 191 | 1 |
| mGdr9-2 | 9 | 342.81 | 67.27 | 121813709 | 138263608 | 1 | 366 | 1 |
| mGdr9-3 | 9 | 406.1 | 41.11 | 140479027 | 143584584 | 2 | 99 | 1 |
| mGdr9-4 | 9 | 545.41 | 9.02 | 152261363 | 152868292 | 1 | 27 | 1 |
| mGdr10-1 | 10 | 191.08 | 74.96 | 11809394 | 88456785 | 1 | 967 | 1 |
| mGwc2-8 | 2 | 688.71 | 27.43 | 240602822 | 240822158 | 2 | 4 | 1 |
| mGwc3-1 | 3 | 54.39 | 37.8 | 2741789 | 4578499 | 1 | 62 | 1 |
| mGwc3-6 | 3 | 786.82 | 54.32 | 227058877 | 233608396 | 1 | 198 | 1 |
| mGwc4-1 | 4 | 187.13 | 59.17 | 17629702 | 26264256 | 2 | 139 | 1 |
| mGwc6-1 | 6 | 32.72 | 30.56 | 2464171 | 6622021 | 1 | 82 | 1 |
| mGwc6-2 | 6 | 88.39 | 14.58 | 35194863 | 59602132 | 1 | 214 | 1 |
| mGwc6-3 | 6 | 121.62 | 10.92 | 82562963 | 91508442 | 1 | 171 | 1 |
| mGwc7-1 | 7 | 71.52 | 8.17 | 5198862 | 6406526 | 1 | 31 | 1 |
| mGwc7-2 | 7 | 101.73 | 10.42 | 8554761 | 9855451 | 4 | 32 | 1 |
| mGwc9-1 | 9 | 82.04 | 80.42 | 7095504 | 15256872 | 1 | 212 | 1 |
| mGwc9-6 | 9 | 505.32 | 21.41 | 148384930 | 150290952 | 2 | 59 | 1 |
| mGwc9-7 | 9 | 618.55 | 21.28 | 156649774 | 157683592 | 2 | 59 | 1 |

**Supplementary Table 2** Comparison of previous and our research results.

| **Co-MQTL** | **Overlap-domain** | **Chr.** | **Position of MQTL in Our results (Mb)** | **Position of MQTL in Sala's results (Mb)** | **Overlap-position (Mb)** |
| --- | --- | --- | --- | --- | --- |
| Co-MQTL1 | mGwc2-3_S10 | 2 | 338.0-356.9 | 330.3-372.8 | 338.0-356.9 |
| Co-MQTL2 | mGwc2-5_S11 | 2 | 405.9-462.8 | 399.2-422.6 | 405.9-422.6 |
| Co-MQTL3 | mGwc2-5_S12 | 2 | 405.9-462.8 | 432.5-526.6 | 432.5-462.8 |
| Co-MQTL4 | mGwc2-6_S12 | 2 | 513.6-518.2 | 432.5-526.6 | 513.6-518.2 |
| Co-MQTL5 | mGwc3-5_S15 | 3 | 450.3-542.2 | 446.7-498.4 | 450.3-498.4 |
| Co-MQTL6 | mGwc3-5_S16 | 3 | 450.3-542.2 | 513.8-635.7 | 513.8-542.2 |
| Co-MQTL7 | mGwc4-2_S17 | 4 | 250.2-253.7 | 243.5-362.2 | 250.2-253.7 |
| Co-MQTL8 | mGwc4-3_S17 | 4 | 262.0-274.4 | 243.5-362.2 | 262.0-274.4 |
| Co-MQTL9 | mGwc5-2_S20 | 5 | 293.2-305.0 | 269.2-323.2 | 293.2-305.0 |
| Co-MQTL10 | mGwc5-4_S21 | 5 | 407.2-455.1 | 445.7-490.7 | 445.7-455.1 |
| Co-MQTL11 | mGwc6-4_S22 | 6 | 155.2-161.3 | 149.2-181.6 | 155.2-161.3 |
| Co-MQTL12 | mGwc6-5_S23 | 6 | 297.5-326.7 | 292.0-485.3 | 297.5-326.7 |
| Co-MQTL13 | mGwc7-3_S24 | 7 | 189.6-190.4 | 190.3-264.6 | 190.3-190.4 |
| Co-MQTL14 | mGwc9-3_S31 | 9 | 231.3-275.1 | 165.8-249.5 | 231.3-249.5 |

**Supplementary Table 3** Comparison of GWAS and our research results.

| **Gene_v4** | **Gene_v3** | **Gene Symbol** | **Pathway** | **MQTL** | **GWAS Traits** |
| --- | --- | --- | --- | --- | --- |
| Zm00001d007181 | GRMZM2G031329 |  | 'signalling.calcium' | mGdr2-4 | GDR |
| Zm00001d002641 |  |  |  | mGwc2-1, mGdr2-1 | GDR |
| Zm00001d005403 |  |  |  | mGdr2-2 | GDR |
| Zm00001d007793 | GRMZM2G083000 |  | 'protein.degradation.ubiquitin.E3.SCF.FBOX' | mGdr2-5 | GDR |
| Zm00001d002535 | GRMZM2G146847 |  | 'protein.degradation.ubiquitin.E3.RING' | mGwc2-1, mGdr2-1 | GDR |
| Zm00001d002462 | GRMZM2G361049 |  | 'protein.degradation.AAA type' | mGwc2-1, mGdr2-1 | GDR |
| Zm00001d041691 |  |  |  | mGdr3-4 | GDR |
| Zm00001d023559 | GRMZM2G046284 | ZmPZB01301 | 'PS.calvin cycle.aldolase' | mGwc10-1 | GWC |
| Zm00001d023565 | GRMZM2G093895 | ZmTCPTF12 | 'RNA.regulation of transcription.TCP transcription factor family' | mGwc10-1 | GWC |
| Zm00001d042378 | GRMZM2G068688 |  | 'protein.degradation' | mGwc3-4 | GWC |
| Zm00001d023563 | GRMZM2G475197 |  | 'signalling.receptor kinases.wall associated kinase' | mGwc10-1 | GWC |
| Zm00001d002641 |  |  |  | mGwc2-1, mGdr2-1 | GWC |
| Zm00001d023564 | GRMZM2G093950 |  | 'RNA.processing' | mGwc10-1 | GWC |
| Zm00001d049557 | GRMZM2G092535 |  | 'RNA.processing' | mGwc4-2 | GWC |
| Zm00001d046583 | GRMZM5G805627 |  | 'protein.synthesis.ribosomal protein.eukaryotic.40S subunit.S14' | mGwc9-3 | GWC |
| Zm00001d023560 | GRMZM2G347226 |  | 'signalling.calcium' | mGwc10-1 | GWC |
| Zm00001d043419 |  |  |  | mGwc3-5 | GWC |
| Zm00001d023562 | GRMZM2G475170 |  | 'stress.biotic' | mGwc10-1 | GWC |
| Zm00001d042442 | GRMZM2G004320 |  | 'protein.postranslational modification' | mGwc3-4 | GWC |
| Zm00001d002462 | GRMZM2G361049 |  | 'protein.degradation.AAA type' | mGwc2-1, mGdr2-1 | GWC |
| Zm00001d043382 | GRMZM2G094712 | ZmGOT1 | 'amino acid metabolism.synthesis.central amino acid metabolism.aspartate.aspartate aminotransferase' | mGwc3-5 | GWC |

**Supplementary Table 4** Candidate genes related to hormones.

| **Gene_v4** | **Gene_v3** | **Hormone** | **Gene Symbol** | **Full Name** | **Homologous Arabidopsis Gene ID** | **Homologous Arabidopsis Gene Symbol** | **Homologous Arabidopsis Gene Full Name** | **MQTLs** |
| --- | --- | --- | --- | --- | --- | --- | --- | --- |
| Zm00001d049277 | GRMZM2G047800 | ABA |  |  | AT3G26760.1 |  |  | mGdr4-1 |
| Zm00001d003512 | GRMZM2G127139 | ABA | ZmZEP1-1 | zeaxanthin epoxidase 1-1 |  |  |  | mGwc2-2 |
| Zm00001d003513 | GRMZM2G127139 | ABA | ZmZEP1-2 | zeaxanthin epoxidase 1-2 | AT5G67030.1 | AtZEP | zeaxanthin epoxidase | mGwc2-2 |
| Zm00001d017199 | GRMZM2G072034 | ABA |  |  | AT5G58070.1 | AtTIL | temperature-induced lipocalin | mGwc5-4 |
| Zm00001d007876 | GRMZM2G408158 | ABA | ZmNCED4 | nine-cis-epoxycarotenoid dioxygenase 4 | AT1G78390.1 | AtNCED9 | nine-cis-epoxycarotenoid dioxygenase 9 | mGdr2-5 |
| Zm00001d023690 |  | ABA | ZmNCED10 | nine-cis-epoxycarotenoid dioxygenase 10 |  |  |  | mGwc10-1 |
| Zm00001d006342 | GRMZM2G081571 | ABA | ZmMOCS2 | molybdenum cofactor sulfurase 2 | AT1G30910.1 |  |  | mGwc2-5 |
| Zm00001d011117 | GRMZM2G363429 | ABA |  |  | AT1G19630.1 | AtCYP722A1 | cytochrome P450, family 722, subfamily A, polypeptide 1 | mGwc8-4 |
| Zm00001d002592 | GRMZM2G164405 | ETH | ZmACS2 | 1-aminocyclopropane  -1-carboxylate synthase 2 | AT4G11280.1 | AtACS6 | 1-aminocyclopropane  -1-carboxylic acid synthase 6 | mGdr2-1, mGwc2-1 |
| Zm00001d045479 | GRMZM2G018006 | ETH | ZmACS3 | 1-aminocyclopropane  -1-carboxylate synthase 3 | AT5G51690.1 | AtACS12 | 1-aminocyclopropane  -1-carboxylate synthase 12 | mGwc9-2 |
| Zm00001d011208 | GRMZM2G013448 | ETH | ZmACCO5 | 1-aminocyclopropane  -1-carboxylate oxidase 5 |  |  |  | mGwc8-4 |
| Zm00001d046848 | GRMZM2G111082 | ETH |  |  | AT2G19590.1 | AtACO1 | ACC oxidase 1 | mGwc9-3 |
| Zm00001d004718 | GRMZM5G854264 | ETH | ZmACCO6 | 1-aminocyclopropane  -1-carboxylate oxidase 6 | AT1G77330.1 |  |  | mGwc2-3 |
| Zm00001d004719 | GRMZM5G854264 | ETH | ZmACCO6 | 1-aminocyclopropane  -1-carboxylate oxidase 6 | AT2G19590.1 | AtACO1 | ACC oxidase 1 | mGwc2-3 |
| Zm00001d042996 |  | ETH |  |  | AT4G38800.1 | AtMTN1 | methylthioadenosine nucleosidase 1 | mGwc3-5 |
| Zm00001d049823 | GRMZM5G896883 | ETH | ZmMTN1 | 5'-methylthioadenosine nuclease 1 | AT4G24340.1 |  |  | mGwc4-3 |
| Zm00001d045558 |  | ETH | ZmMTN2 | 5'-methylthioadenosine nuclease 2 | AT1G77670.1 |  |  | mGwc9-2 |
| Zm00001d013992 | GRMZM2G174145 | ETH | ZmTIDP3358 |  | AT4G39640.1 | AtGGT1 | gamma-glutamyl transpeptidase 1 | mGwc5-1 |
| Zm00001d003446 | GRMZM2G065073 | ETH | ZmIDP2449 |  | AT2G46370.4 | AtJAR1 | auxin-responsive GH3 family protein | mGwc2-2 |
| Zm00001d009714 | GRMZM2G162413 | ETH | ZmAAS11 | auxin amido synthetase 11 | AT5G19040.1 | AtIPT5 | isopentenyltransferase 5 | mGwc8-2 |
| Zm00001d049601 | AC196412.3_FG001 | IAA |  |  | AT2G20340.1 |  |  | mGdr4-2, mGwc4-2 |
| Zm00001d041648 |  | IAA |  |  |  |  |  | mGdr3-4 |
| Zm00001d005602 |  | IAA |  |  |  |  |  | mGdr2-2 |
| Zm00001d005439 | GRMZM2G333478 | IAA | ZmYUC9 | yucca 9 | AT1G04610.1 | AtYUC3 | yucca 3 | mGdr2-2 |
| Zm00001d023718 | GRMZM2G091819 | IAA | ZmDE18 | defective 18 | AT1G21430.1 | AtYUC11 | flavin-binding monooxygenase family protein | mGwc10-1 |
| Zm00001d004467 | GRMZM2G141383 | IAA | ZmYUC4 | yucca 4 | AT4G28720.1 | AtYUC8 | flavin-binding monooxygenase family protein | mGwc2-3 |
| Zm00001d007395 | GRMZM2G410567 | IAA | ZmAAS3 | auxin amido synthetase 3 | AT1G28130.1 | AtGH3.17 | auxin-responsive GH3 family protein 17 | mGwc2-7 |
| Zm00001d043244 | GRMZM2G061515 | IAA | ZmAAS6 | auxin amido synthetase 6 | AT5G54510.1 | AtGH3.6 | auxin-responsive GH3 family protein 6 | mGwc3-5 |
| Zm00001d043350 | GRMZM2G033359 | IAA | ZmAAS7 | auxin amido synthetase 7 | AT4G37390.1 | AtGH3.2 | auxin-responsive GH3 family protein 2 | mGwc3-5 |
| Zm00001d043235 | GRMZM2G022934 | IAA |  |  | AT2G23620.1 | AtMES1 | methyl esterase 1 | mGwc3-5 |
| Zm00001d007180 | GRMZM2G031125 | GA | ZmGA13ox1 | gibberellin 13-oxidase 1 | AT5G24910.1 | AtCYP714A1 | cytochrome P450, family 714, subfamily A, polypeptide 1 | mGdr2-4 |
| Zm00001d045563 | GRMZM2G093195 | GA | ZmD3 | dwarf plant 3 | AT2G32440.1 | AtKAO2 | ent-kaurenoic acid hydroxylase 2 | mGwc9-2 |
| Zm00001d043411 | GRMZM2G022679 | GA | ZmGA2ox3 | gibberellin 2-oxidase 3 | AT1G78440.1 | AtGA2OX1 | gibberellin 2-oxidase 1 | mGwc3-5 |
| Zm00001d017294 | GRMZM2G153359 | GA | ZmGA2ox4 | gibberellin 2-oxidase 4 | AT4G21200.1 | AtGA2OX8 | gibberellin 2-oxidase 8 | mGwc5-4 |
| Zm00001d040810 | GRMZM2G436770 | CTK | ZmIPT7 | isopentenyl transferase 7 | AT4G35190.1 |  |  | mGwc3-3 |
| Zm00001d021450 | GRMZM2G019363 | CTK |  |  | AT5G06300.1 |  |  | mGwc7-4 |
| Zm00001d011246 | GRMZM5G842645 | CTK |  |  | AT1G75450.1 | AtCKX5 | cytokinin oxidase 5 | mGwc8-4 |
| Zm00001d043293 | GRMZM5G817173 | CTK | ZmCKO4 | cytokinin oxidase 4 | AT2G36750.1 | AtUGT73C1 | UDP-glucosyl transferase 73C1 | mGwc3-5 |
| Zm00001d007378 | GRMZM2G056335 | CTK |  |  | AT3G22250.1 |  |  | mGwc2-7 |
| Zm00001d017326 | GRMZM2G159918 | CTK |  |  | AT5G14860.1 |  |  | mGwc5-4 |
| Zm00001d046479 | AC234524.1_FG005 | CTK |  |  | AT2G36800.1 | AtDOGT1 | don-glucosyltransferase 1 | mGwc9-3 |
| Zm00001d049292 | GRMZM2G389944 | BR |  |  | AT2G36800.1 | AtDOGT1 | don-glucosyltransferase 1 | mGdr4-1 |
| Zm00001d049282 | GRMZM2G007012 | BR |  |  |  |  |  | mGdr4-1 |
| Zm00001d011029 |  | BR |  |  |  |  |  | mGwc8-4 |
| Zm00001d006425 |  | BR |  |  | AT1G17420.1 | AtLOX3 | lipoxygenase 3 | mGwc2-5 |
| Zm00001d003533 | GRMZM2G104843 | JA | ZmTS1 | tassel seed 1 | AT3G25760.1 | AtAOC1 | allene oxide cyclase 1 | mGwc2-2 |
| Zm00001d047340 | GRMZM2G415793 | JA |  |  | AT1G76690.1 | AtOPR2 | 12-oxophytodienoate reductase 2 | mGwc9-4 |
| Zm00001d011097 |  | JA | ZmOPR4 | 12-oxo-phytodienoic acid reductase 4 | AT1G76680.1 | AtOPR1 | 12-oxophytodienoate reductase 1 | mGwc8-4 |
| Zm00001d003584 | GRMZM2G087192 | JA | ZmOPR5 | 12-oxo-phytodienoic acid reductase 5 |  |  |  | mGwc2-2 |
| Zm00001d040842 | GRMZM2G068947 | JA | ZmOPR6 | 12-oxo-phytodienoic acid reductase 6 | AT4G16760.1 | AtACX1 | acyl-CoA oxidase 1 | mGwc3-3 |
| Zm00001d045606 | GRMZM2G052389 | JA | ZmUMC2337 |  | AT2G46370.4 | AtJAR1 | auxin-responsive GH3 family protein | mGwc9-2 |
| Zm00001d009714 | GRMZM2G162413 | JA | ZmAAS11 | auxin amido synthetase 11 | AT3G06350.1 | AtMEE32 |  | mGwc8-2 |
| Zm00001d006242 | GRMZM2G573867 | SA |  |  | AT5G66120.2 |  |  | mGwc2-5 |
| Zm00001d041700 | GRMZM5G804881 | SA |  |  | AT2G21940.4 | AtSK1 | shikimate kinase 1 | mGdr3-4 |
| Zm00001d052247 | GRMZM2G161566 | SA |  |  | AT1G08250.1 | AtADT6 | arogenate dehydratase 6 | mGdr4-4 |
| Zm00001d005351 | GRMZM2G466543 | SA |  |  | AT3G06350.1 | AtMEE32 |  | mGdr2-2 |
| Zm00001d040850 | GRMZM2G314652 | SA | ZmCL40355_1 |  |  |  |  | mGwc3-3 |
| Zm00001d042538 |  | SA |  |  | AT2G37040.1 | AtPAL1 | PHE ammonia lyase 1 | mGwc3-4 |
| Zm00001d017274 | GRMZM2G074604 | SA | ZmPAL1 | phenylalanine ammonia lyase homolog 1 |  |  |  | mGwc5-4 |
| Zm00001d017279 | GRMZM2G170692 | SA | ZmPAL7 | phenylalanine ammonia lyase 7 | AT2G37040.1 | AtPAL1 | PHE ammonia lyase 1 | mGwc5-4 |
| Zm00001d017276 | GRMZM2G334660 | SA | ZmPAL8 | phenylalanine ammonia lyase 8 | AT2G37040.1 | AtPAL1 | PHE ammonia lyase 1 | mGwc5-4 |
| Zm00001d017275 | GRMZM2G029048 | SA | ZmPAL9 | phenylalanine ammonia lyase 9 | AT3G53260.1 | AtPAL2 | phenylalanine ammonia-lyase 2 | mGwc5-4 |
| Zm00001d045679 |  | SA |  |  | AT1G44170.1 | AtALDH3H1 | aldehyde dehydrogenase 3H1 | mGwc9-2 |
| Zm00001d004731 | GRMZM2G118800 | SA | ZmALDH13 | aldehyde dehydrogenase 13 | AT3G29200.1 | AtCM1 | chorismate mutase 1 | mGwc2-3 |
| Zm00001d023580 | GRMZM2G103546 | SA | ZmALDH25 | aldehyde dehydrogenase 25 | AT1G23800.1 | AtALDH2B7 | aldehyde dehydrogenase 2B7 | mGwc10-1 |
| Zm00001d045706 | GRMZM2G058675 | SA | ZmRF2 | restorer of fertility 2 | AT1G44170.1 | AtALDH3H1 | aldehyde dehydrogenase 3H1 | mGwc9-2 |
| Zm00001d043356 | GRMZM2G028369 | SA | ZmCMU3 | chorismate mutase 3 | AT2G22250.2 | AtMEE17 |  | mGwc3-5 |
| Zm00001d043382 | GRMZM2G094712 | SA | ZmGOT1 | glutamate-oxaloacetate transaminase 1 | AT1G08250.1 | AtADT6 | arogenate dehydratase 6 | mGwc3-5 |
| Zm00001d010190 | GRMZM2G033799 | SA | ZmGOT5 | glutamate-oxaloacetate transaminase 5 | AT5G11520.1 | AtASP3 | aspartate aminotransferase 3 | mGwc8-3 |
| Zm00001d025333 | GRMZM2G125923 | SA |  |  |  |  |  | mGwc10-2 |
| Zm00001d046750 |  | SL |  |  | AT1G10830.1 | AtZ-ISO | 15-cis-zeta-carotene isomerase | mGwc9-3 |
| Zm00001d023655 | GRMZM2G011746 | SL | ZmY9 | pale yellow 9 |  |  |  | mGwc10-1 |
